# Extracellular vesicles from IPFP-MSCs trigger osteoarthritis by transferring mtDNA

**DOI:** 10.1016/j.bioactmat.2025.11.046

**Published:** 2025-12-11

**Authors:** Shiyu Li, Zi Yan, Xinwang Zhi, Weihan Zheng, Ziqi Zhang, Zhenning Dai, Wanying Chen, Hui Lu, Ziyi Feng, Ting Cheng, Wenhui Liu, Baoyu Sun, Yuhai Ma, Bing Zhang, Jianyuan Zhao, Han Liu, Jiacan Su

**Affiliations:** a Department of Orthopedics, Xinhua Hospital, Shanghai Jiao Tong University School of Medicine, Shanghai, 200092, China; b Department of Immunology, Institute of Geriatric Immunology, School of Medicine, Jinan University, Guangzhou, 510632, China; c Institute of Translational Medicine, Shanghai University, Shanghai, 200444, China; d Department of Urology, Sun Yat-sen Memorial Hospital, Sun Yat-sen University, Guangzhou, 510120, China; e Department of Pediatric Orthopedics, Department of Pediatric Surgery, Guangzhou Women and Children's Medical Center, Guangzhou Medical University, Guangzhou, 511436, China; f The First Affiliated Hospital of Jinan University, Jinan University, Guangzhou, 510632, China; g Biliary and Pancreatic Surgery of the First Affiliated Hospital of Guangzhou University of Chinese Medicine, Guangzhou, 510405, China; h Department of Stomatology, Guangdong Provincial Key Laboratory of Research and Development in Traditional Chinese Medicine, Guangdong Second Traditional Chinese Medicine Hospital, Guangzhou, 510095, China; i School of Stomatology, Jinan University, Guangzhou, 510632, China; j Engineering Research Center of Techniques and Instruments for Diagnosis and Treatment of Congenital Heart Disease, Institute of Developmental and Regenerative Medicine, Shanghai Jiao Tong University School of Medicine, Shanghai, 200092, China; k Ministry of Education-Shanghai Key Laboratory of Children's Environmental Health, Institute for Developmental and Regenerative Cardiovascular Medicine, Xinhua Hospital, Shanghai Jiao Tong University School of Medicine, Shanghai, 200092, China; l Suzhou Innoovation Center of Shanghai University, Suzhou, 215000, China; m Sanming Second Hospital, Sanming, 366000, China

**Keywords:** Osteoarthritis, Extracellular vesicles, Mitochondrial dysfunction, Mitochondria-derived vesicles, Infrapatellar fat pad, cGAS-STING, mtDNA

## Abstract

Infrapatellar fat pad mesenchymal stem cells (IPFP-MSCs) extracellular vesicles (EVs) are found to be capable of accelerating Osteoarthritis (OA) progression. However, which pathways and which pathogenic EVs subgroups are involved are not defined. In our study we found that there were a higher percentage of TOMM20^+^ EV's within the total synovial fluid EV's from OA patients than from trauma patients as well as increased mtDNA content. This implicates the mitochondria derived EV sub-group - mitochondria derived vesicles (MDVs) as a potential driver in OA. We found with the single-cell data that MDVs may be secreted from IPFP-MSCs with VPS35. Furthermore, these cells were harvested from the body of the OA patient. IPFP-MSC derived MDVs can deliver exogenous mtDNA to chondrocytes by fusing directly, thus inhibiting chondrocyte matrix synthesis, inducing mitochondrial dysfunction, and activating pro-inflammatory signaling cascades in chondrocytes. Protein microarrays showed that MDVs delivered exogenous mtDNA to chondrocytes, which then activated the cGAS-STING pathway and downstream inflammatory mediators (TBK1, NF-κB, TNF-α). Intra-articular MDV injection worsened cartilage degradation and synovitis in OA rats but STING inhibition alleviated them. This study showed that IPFP-MSC-derived MDVs are essential for OA pathogenesis via mtDNA transfer and cGAS-STING pathway activation. These results show how the mitochondria and immune system talk to each other in the joints causing pain and destroying the cartilage, MDVs are new things that can tell us if someone has this disease and help doctors fix it. Pharmacological blockade of the cGAS-STING axis has shown therapeutic potential, providing a dual approach to mitigate mitochondrial stress and innate immune hyperactivation in OA.

## Highlights

• OA synovial fluid is enriched in MDVs derived from IPFP-MSCs.
• OA-MDVs activate the mtDNA–cGAS–STING pathway in chondrocytes.

## Graphical abstract

**Schematic diagram.**MDVs from IPFP-MSCs bring exogenous mtDNA to chondrocytes, turn on the cGAS-STING pathway, start an immune response, and make OA inflammation bigger.

**Figure:**
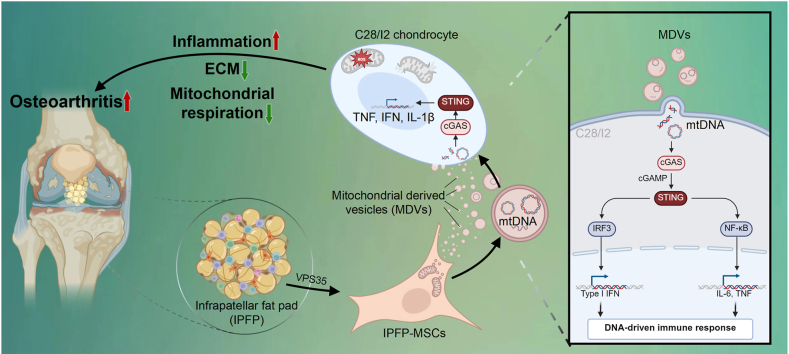

## 1 Introduction

Knee osteoarthritis (OA) is a common chronic joint condition. It has as characteristic pathologic features the progressive deterioration of articular cartilage, the inflammatory reaction in the synovium and the alteration of the underlying subchondral bone. Together, these abnormal changes cause the usual symptoms of pain and problems with joint function [1]. Though we lack a full understanding of the causes of OA, it is believed to involve a combination of mechanical, cellular, and inflammatory factors. Infrapatellar fat pad (IPFP) has been reclassified from a structural element to a potential contributor to OA pathogenesis [2,3]. The IPFP is filled with blood vessels, nerves, and mesenchymal stem cells (IPFP-MSCs) that contribute to joint lubrication and cushioning. In OA, inflammation within the IPFP is thought to cause pain by inducing angiogenesis [4,5]. The release of extracellular vesicles (EVs) by IPFP-MSCs constitutes a conceivable route toward the mitigation of OA advancement. EVs are of notable interest in scientific circles for their critical parts in facilitating cellular correspondence and modifying disease conditions [6,7].

EVs are subcellular, lipid bilayer enclosed, nanoscale sized structures [8]. Mediate key cell signal transmission pathways with a large volume of goods delivery system, carrying out transmission of goods like proteins, lipids and nucleic acids [9,10]. In terms of OA, EVs from IPFP-MSCs have attracted a lot of attention for their ability to affect the course of the disease [11,12]. With recent studies showing that they can promote the development of OA by causing metabolic dysfunction and fibrosis of chondrocytes, which are key events in the pathogenesis of OA. Inflammatory conditions, IPFP-MSC derived EVs may contribute to the disease by promoting cartilage destruction and inflammation. These are not good things, but there is some evidence that these EVs might have some healing power, and some studies suggest they could be used to help bodies grow new parts [13].EVs are considered to contain different subpopulations like apoptotic bodies, microvesicles (MVs), exosomes (EXOs), and mitochondria-derived vesicles (MDVs) [14,15]. But the particular functions of these EV subtypes in OA development are poorly known. There is a gap in knowledge when it comes to how different kinds of electric vehicle users take part in OA development and if we can use them as therapy targets [16,17].

Molecular regulation of OA is also affected by the cGAS-stimulator of interferon genes(STING) pathway. Cytosolic DNA receptor cGAS recognizes double stranded DNA (dsDNA) which leads to activation of STING [18,19]. Evidences show that the abnormal mechanical stress environment in OA leads to damage of chondrocyte nucleus, which causes the cGAS-STING axis to be activated by the presence of dsDNA in the cytoplasm, which serves as a danger-sensing mechanism [20,21]. And such activation triggers a downstream signaling cascade by interferon regulatory factor 3 (IRF3), TBK1 and NF-κB to synthesize inflammatory factors. While endogenous DNA driven cGAS-STING activation is known, it is not clear whether exogenous DNA drives cGAS-STING in osteoarthritis. Studying in association to these conditions confirming extracellular dsDNA and circulating mtDNA are activating of the pathway in other pathological conditions as well [22]. For example, in neurodegenerative disease, mitochondrial cytosolic dsDNA has been implicated in inducing cytotoxicity and neurodegeneration [23]. In addition to the OA pathogenesis, experimental evidence shows mtDNA-mediated stimulation of the OA pathway in both acute and chronic renal disorders [24,25]. Whether exogenous DNA activates cGAS-STING in OA and if EVs are involved in the process is yet to be completely answered. Further investigation is necessary.

We showed quantitatively high level of MDVs in the EV fractions isolated from synovial fluids of osteoarthritic joints. We assume that MDVs released by IPFP-MSCs bring mtDNA into chondrocytes and function as an exogenous DNA trigger of the cGAS-STING pathway. This activation may boost the inflammatory cascade, making the development of OA worse. Our results imply an interesting way that EVs - especially MDVs - could play a part in OA disease and might be a spot to try to change the course of the illness.

## 2 Materials and methods

### 2.1 Collection of synovial fluid and IPFP tissues from patients with OA

Patient recruitment, tissue collection, and isolation of primary IPFP-MSCs are in line with our existing protocol [26]. All the clinical specimen collections received prior approval from the Institutional Review Board of Guangzhou Women and Children's Medical Center, and they were carried out according to the informed consent of the participants. Synovial fluid and IPFP tissues from patients with OA were obtained during total joint replacement procedures at the Guangzhou Women and Children's Medical Center (n = 8). Control samples, including normal synovial fluid and IPFP tissues, were collected from patients with trauma with no known history of OA or rheumatoid arthritis (n = 21), as in Table S1.

### 2.2 ELISA detection

For inflammatory protein biomarkers, synovial fluid samples were obtained via needle aspiration. To clarify the samples, Synovial fluid was digested with 2 mg/mL hyaluronidase (Sigma) for 30 min at 37 °C, centrifuged at 14,000×*g* for 20 min, and supernatants stored at −80 °C. C-reactive protein (CRP; JL13865-48T, J&L Biological, China), matrix metalloproteinase-13 (MMP13; EH0234-96T, FineTest, China), and interleukin-1β (IL-1β; SJIM-1236H, JINGMEI, China) were selected as biomarkers for ELISA testing. Standard curves were generated through serial dilutions and quantified by microplate perimetry EVs. After incubation of the test samples with the working solution, standard curve interpolation was employed to determine protein concentrations.

For EVs and MDVs samples, 1 mg/mL EVs were used for ELISA detection. Translocase of outer mitochondrial membrane 20 (TOMM20; JM-0803H2, JINGMEI, China) and transcription factor A of mitochondrial (TFAM, MM-51486H1, MEIMIAN, China) were detected, and the ΔCt values of mtDNA *ND1*, *ND2*, *COX1* and *ATP6* were linearly fitted to calculate the Pearson correlation coefficient (R values) and P values using GraphPad Prism v10.0.The same method was used to detect inflammatory proteins in the synovial fluid of rats *in vivo* experiments, utilizing Rat Interleukin 1β (IL-1β; SP12225, Spbio, China) and Rat Interleukin 6 (IL-6, SP12279, Spbio, China).

### 2.3 Isolation of extracellular vesicles

Using standard differential centrifugation, EVs were harvested from samples of synovial fluid [27]. First, the synovial fluid was centrifuged at 300×*g* for 10 min under refrigerated conditions. The supernatant was then transferred to new tubes and centrifuged at 2000×*g* for 30 min under refrigerated conditions. The clarified supernatant underwent secondary centrifugation at 10,000×*g* for 30 min under refrigerated conditions. Subsequently, the supernatant was aliquoted into ultracentrifuge tubes and subjected to a 70 min spin at 100,000×*g* under conditions of 4 °C. EV pellets were collected following supernatant aspiration. These were reconstituted in 5 mL PBS prior to terminal centrifugation at 100,000×*g* for 70 min under refrigeration. Finally, the supernatant was discarded, and the EV-containing pellet was resuspended in a desired volume of PBS for downstream analysis or stored at −80 °C.

### 2.4 Nanoparticle tracking analysis (NTA)

NTA (ZetaView Instruments, Particle Metrix, Meerbusch, Germany) was performed to measure the particle size of EVs and MDVs. Quantification was performed using NTA software (Particle Metrix).

### 2.5 Morphological and lamellarity characterization of EVs and MDVs

Mitochondrial ultrastructure and the presence of EVs and MDVs were analyzed by Transmission Electron Microscopy (TEM) and Cryo-transmission electron microscopy (cryo-TEM). Following 2 min glow-discharge activation, carbon-coated copper grids received 5 μL aliquots of specimen. Subsequently, 5 μL of 2 % uranyl acetate stain (freshly prepared) was applied for 2 min incubation. Filter paper blotting removed excess material, yielding a thin stained film. The staining and blotting procedure was repeated using 2 % uranyl acetate, and the grid was allowed to air-dry prior to imaging. TEM micrographs were acquired using a Tecnai T12 TEM microscope operated at 100 kV.

MDV morphology was also examined using cryo-TEM (Tecnai G2 F20), with samples prepared by vitrification (FEI, Hillsboro, OR), to confirm the presence of a bilayer membrane and spherical structure.

### 2.6 Bicinchoninic acid (BCA) assay

By using BCA Protein Quantitation Assay Kit (P0011, Beyotime, China), we quantified EV protein concentrations of human synovial fluid samples and MDVs from culture medium, according to standard protocols.

### 2.7 Western blotting (WB)

To detect the specific protein biomarkers of EVs, WB analysis was performed. To normalize for lack of vesicle specific loading control, all samples were normalized to total protein content of BCA assay. Proteins were isolated with ice-cold RIPA buffer containing a protease inhibitor cocktail (P0013K, Beyotime, China). After measuring protein concentrations, equal quantities (generally 20–30 μg per lane) were separated by electrophoresis on 10 % SDS-polyacrylamide gels and then transferred electrically to nitrocellulose membranes. Subsequent to a 1 h room-temperature block in 5 % skim milk diluted in TBST, the membranes were incubated overnight with specific primary antibodies at 4 °C. Antigens bound to the membranes were visualized by treating with horseradish peroxidase (HRP)-conjugated secondary antibodies for 60 min at room temperature, followed by chemiluminescent detection. Details of all antibodies applied are provided in Table S2. An enhanced chemiluminescence substrate (P0018HM, Beyotime, China) was used for protein detection, and band intensity was quantified relative to β-actin as an internal control.

### 2.8 Nanoscale flow cytometry (NanoFCM)

EVs isolated from synovial fluid were analyzed for protein expression profiles using nanoFCM with a NanoAnalyzer instrument (NanoFCM Inc., Nottingham, UK). The system was equipped with dual excitation lasers at 488 nm for FITC and 647 nm for Alexa Fluor 647 detection. EVs (5 × 10^5^ particles) were stained with FITC-conjugated anti-CD63 (1 ng/μL; ab21851, Abcam, UK) and Alexa Fluor 647-conjugated anti-TOM20 (1 ng/μL; sc-17764, Santa Cruz Biotechnology, USA) in 50 μL PBS, along with isotype-matched IgG controls. Before staining, the antibody solutions were spun down at 12,000×*g* for 10 min to remove aggregates. After 90 min of incubation with constant agitation at 25 °C, labeled EVs were pelleted via ultracentrifugation (120,000×*g*, 70 min) and resuspended in PBS at a 1:100 dilution for nanoFCM. This protocol allowed for quantification of EV subpopulations by surface protein expression. For analysis, the total number of EVs in each sample of synovial fluid was quantified as well as the percentage of positively stained particles.

### 2.9 mtDNA Copy Number Assay

The mtDNA copy numbers present in IPFP-MSCs, EVs and MDVs were compared using standardized qPCR with SYBR green detection and absolute quantification relative to validated standards [28].

For IPFP-MSCs, total DNA was extracted, and relative mtDNA copy number was quantified by qPCR with ND1, ND2, COX1, and ATP6 specific primers, normalized to nuclear *β-globin*.

EVs and MDVs total RNA isolation was carried out using the TRIzol reagent (TRIzol Reagent 15596026CN,Invitrogen, USA), and measured through a Multiskan Go spectrophotometer system (Thermo Fisher Scientific, USA). Reverse transcribed RNA into cDNA by PrimeScript RT Master Mix (RR036A, Takara, Japan). Quantitative PCR was performed using SYBR Premix Ex *Taq*II (RR820A, Takara, Japan) according to the manufacturer's instructions. Each sample was run 3 times in triplicate.

The ΔCt method was applied to compute relative mtDNA expression, with results expressed as fold changes compared to the control group. Primer sequences are provided in Table S3.

### 2.10 Cell culture

IPFP-MSCs were isolated from IPFP tissues using a previously described enzymatic digestion method [29], and were designated as WT IPFP-MSCs or OA IPFP-MSCs. IPFP-MSCs were maintained in DMEM/F-12 basal medium (C11330500BT, Gibco, USA) containing 10 % exosome-depleted fetal bovine serum (EXO-FBS-50A, SBI, USA) and 1 % penicillin-streptomycin antibiotic solution (10,000 U/mL; 15140122, Gibco,USA). Immunofluorescence (IF) staining was used to identify the surface protein CD105. The antibody used is listed in Table S2. C28/I2 cells were cultured in DMEM supplemented with 10 % exosome-depleted fetal bovine serum and 1 % penicillin-streptomycin. All cultures were maintained at 37 °C in a 5 % CO_2_-humidified incubator following standard mammalian cell culture protocols.

### 2.11 Purification of MDVs

MDVs were purified as previously described by D'Acunzo [30]. IPFP-MSCs were cultured in T25 flasks until the cell density reached approximately 80 %. Cells were cultured in complete medium with 10 % EXO-free FBS for 48 h. EVs were then isolated from conditioned medium via differential centrifugation. The resulting crude EV pellet was resuspended in 1.5 mL of ice-cold 40 % (w/v) iodixanol solution. Subsequently, a discontinuous iodixanol density gradient was prepared by slowly layering 800 μL of iodixanol solutions at concentrations of 40 %, 20 %, 15 %, 13 %, 11 %, 9 %, and 7 % into an ultracentrifugation tube. The tube was maintained at 4 °C throughout the procedure. A 2 mL volume of EV solution resuspended in PBS was added. Density gradient separation by ultracentrifugation at 200,000×*g* for 16 h on refrigeration. 1.25 mL fraction (fraction 1) was discarded, and then 1.5 mL fractions (fractions 2–6 and 8) were removed from the iodixanol interface. Fraction 7 (20 % iodixanol) was diluted 1:4 (volume) with PBS at 4 °C and was split equally between two 10 ml tubes. After ultracentrifugation (100,000×*g*, 70 min, 4 °C), the supernatant was carefully aspirated from each tube and 500 μL of the remaining volume was kept. The dual pellet fractions were pooled and gently resuspended in PBS to a final volume of 10 mL and spun at the same conditions as above. The pellets were removed after supernatant removal and then dried by air-drying at 37 °C. Fraction 7 was then reconstituted in 1 mL of ice-cold PBS and stored at −80 °C for downstream analysis.

### 2.12 MDV internalization

The PKH26 fluorescent dye (1 μL) was homogenized with 9 μL of Diluent C to prepare the labeling solution. incubation in MDV suspensions at 20–25 °C with protection against LPS, 10 min. The staining was terminated using 1 mL of ice-cold PBS. Stained MDVs were then collected via centrifugation at 100,000×*g* for 20 min at 4 °C. After removal of supernatant the pellet is resuspended in PBS (sterile). This washing process has been performed two times, and the final MDV preparation was diluted in 100 μL of PBS. The stained MDVs (12 μg/mL) were co-incubated with C28/I2 cells pre-labeled with dioctadecyloxacarbocyanine (DiO; 5 mM in DMSO, staining at 37 °C for 20 min) for 4 d. Following incubation, the residual staining solution was aspirated and specimens underwent triple washing with PBS. Cellular fixation was performed using 4 % paraformaldehyde (PFA) for 30 min at room temperature, followed by three additional PBS washes. Nuclei were contrast-labeled with DAPI for 5 min before final mounting.

The uptake inhibitors Dynasore (20 μM; HY-15304, MedChemExpress, USA), Cytochalasin D (5 μM; HY-N6682, MedChemExpress, USA), Amiloride (200 μM; MK-870, MedChemExpress, USA), and Z-Phe-Phe-Phe-OH (20 μM; GA23911, GlpBio, USA), each targeting distinct uptake pathways-clathrin-mediated endocytosis, macropinocytosis, phagocytosis, and membrane fusion respectively, were individually introduced into the culture medium of C28/I2 cells. MDVs and cells were labeled with covalently bound dyes,100 μg MDVs were labeled with 5 μL EvLINK 505 (EL012100201, TINGO, China) following the manufacturer's protocol and incubated with gentle mixing at room temperature in the dark for 30 min. To remove excess dye, the green fuorescently labeled MDVs were ultraflteredat 12000×*g* for 15 min using a 100 kDa ultrafltration unit (UFC910096, Millipore, USA). The purifed sample was collected for subsequent experiments to assess MDV uptake by chondrocytes. C28/I2 cells were seeded into confocal dishes and cultured for 24 h. Subsequently, cell were incubated with EvLINK 505-labeled MDVs for 72 h and washed with PBS. To see the cell membrane, chondrocytes were marked with CellLINK 555 (CL012100221, TINGO, China), then placed in darkness and left at room temperature for 30 min. Cells were washed with PBS and fixed with 4 % PFA for 30min. Nuclei stained with DAPL for 5 min.

Processed samples were imaged by LSM880 (Zeiss, Germany) laser scanning confocal microscopy. And the obtained images were quantified using Image J software to measure the relative fluorescent intensity.

### 2.13 Single-cell sequencing and data analysis

Secondary data analysis was conducted on genomic datasets. This investigation utilized publicly available single-cell and single-nucleus transcriptomic datasets (scRNA-seq and snRNA-seq respectively) retrieved from the NCBI Gene Expression Omnibus database (Accession: GSE216651). Subsequent processing was done according to the Single-cell Best Practices. These datasets are from the team of Ding and Qin. The dataset included nine human donors, including four healthy WT-donors that were processed for scRNA-seq and one that was processed for snRNA-seq, and five OA patients that were processed for scRNA-seq and two for snRNA-seq. All sequencing were done on 10x Genomics Chromium. Integration of the scRNA-seq (34,982 cells) and snRNA-seq (10,206 cells) datasets produced a cellular atlas of 90,389 cells. Split-UMAP to reduce dimensions and have segregated manifold projections, and FindCluster for a de-novo cell classification, unsupervised in 12 different transcriptionally separated clusters.

We investigated the expression levels of key mediators of MDV biogenesis such as CD38, RAB9A, Parkin RBR E3 ubiquitin protein ligase (*PRKN*), retromer complex component VPS35, syntaxin 17 (*STX17*), Toll-interacting protein (*TOLLIP*), dynamin 1-like (DNM1L), sorting nexin 9 (*SNX9*), OPA1 mitochondrial dynamin-like GTPase (*OPA1*) and Ras homolog family member T1 (*RHOT1*). These molecular markers were evaluated across all clusters and compared between the WT and OA cohorts to identify disease-associated transcriptional changes in mitochondrial quality control pathways.

### 2.14 Co-culture of MDVs and chondrocyte pellets

To determine how functional WT-MDVs and OA-MDVs were on C28/I2 cells, chondrogenic differentiation was conducted. The cells in the IL-1β group were cultured in recombinant human IL-1β (10 ng/mL) for 24 h, while the control, WT-MDV, and OA-MDV groups were cultured in proliferation medium. MDVs in both the WT-MDV and OA-MDV groups were also set to 12 μg/mL, the same concentration level as that in OA synovial fluid.

C28/I2 cells (1 × 10^6^/mL) were prepared as a single-cell suspension, centrifuged at 200×*g*, and cultured in 15 mL centrifuge tubes with loosened caps for 7 d to promote initial chondrocyte pellet formation. Following this, the cells were treated according to their group conditions, and complete medium replacement was performed at 72-h intervals during the cultivation period. After 28 d of chondrogenic induction, all samples were fixed, embedded in paraffin, and sectioned. The sections were stained with antibodies against aggrecan, collagen type II (COL2), and SOX9 and incubated at 4 °C for 10 h. Goat anti-rabbit IgG H&L (HRP) secondary antibody was added and incubated for 1 h at room temperature. Immunohistochemical (IHC) staining was performed using a 3,3′-diaminobenzidine tetrahydrochloride kit (P0202; Beyotime, China), followed by hematoxylin counterstaining and mounting in neutral resin. Alcian Blue staining (Morphisto, Offenbach am Main, Germany) of the chondrocyte pellets was also performed according to the manufacturer's protocol. Three random imaging fields were analyzed using an Olympus CKX31 inverted microscope. All antibodies employed in this study are comprehensively itemized in Supplementary Table S2.

### 2.15 Chondrogenic biomarker analysis

Expression of genes associated with chondrogenic differentiation (*aggrecan*, *COL2*, and *SOX9*) was detected using qPCR, as described in Section mtDNA Copy Number Assay. Primers used are provided in Table S2. *GAPDH* was used as an endogenous control.

### 2.16 Protein mass spectrometry analysis

Aliquots of WT-MDVs and OA-MDVs (500 μg/mL) were incubated in 1.5 mL tubes with lysis buffer containing 1 % SDC and protease inhibitors. After sonication, the mixtures were centrifuged at 12,000×*g* for 10 min (4 °C). Equivalent protein quantities from supernatants were subjected to trypsin-mediated digestion, preceded by disulfide reduction using DTT and cysteine alkylation via IAA. Proteolysis was carried out through prolonged incubation overnight, followed by an additional 4-h enzymatic treatment. The resulting peptides were resuspended in solvent A (0.1 % formic acid, 2 % acetonitrile) and fractionated on a NanoElute UHPLC platform. A linear gradient increasing from 6 % to 80 % of solvent B (0.1 % formic acid in acetonitrile) was implemented over 15 min, maintaining a 500 nL/min flow rate. Ionization was performed with a capillary source, and MS analysis was conducted on a timsTOF Pro 2 instrument. Acquisition operated in dia-PASEF mode, covering an *m/z* range of 300–1500 for MS, accompanied by 20 PASEF cycles targeting fragment ions from 400 to 850 *m/z*. All proteomic profiling was performed by Novogene Co., Ltd. (Guangzhou, China).

### 2.17 ROS and mtROS detection assay

C28/I2 cells were plated in confocal imaging dishes at a density of 1 × 10^5^ cells/dish and exposed to experimental conditions for 72 h. Intracellular reactive oxygen species (ROS) levels were assessed using 2′,7′-dichlorodihydrofluorescein diacetate (H_2_DCFDA; Beyotime Biotechnology, China). The fluorescent probe stock solution was diluted 1:1000 in serum-free medium according to manufacturer specifications and incubated with cells for 30 min at 37 °C, followed by two washes with serum-free basal medium to remove residual probe. Nuclei were counterstained with Hoechst under identical conditions.

MitoSOX red (MitoSO™ Red; S0061S, Beyotime, China), a fluorescent probe for detecting mitochondrial superoxide, was used in this experiment. C28I2 cells were plated onto confocal dishes and allowed to adhere overnight. After 72 h of exposure to MDVs diluted in DMEM, the cells were washed three times with PBS. Subsequently, a staining solution containing MitoSOX red and Hoechst was applied, followed by a 15-min incubation period.

### 2.18 Mitochondrial membrane potential and morphology analysis

Changes in Δψm (mitochondrial membrane potential) were measured by the JC-1 dye fluorescence transition (red/green emission ratio) with commercial reagents (Beyotime). MitoTracker Green 1 mM (Beyotime) was prepared in anhydrous DMSO and kept at −20 °C. Cell (1 × 105/well) was seeded into the confocal dishes and cultured for 24h, and stained with prewarmed MitoTracker Green for 2 h. We then viewed our mitochondrial networks on a confocal microscope with the Zess LSM 800 confocal microscope after completing the stains, switching them out. Also ImageJ was used to measure the red/green ratio, and network complexity and branch length.

### 2.19 Seahorse analysis oxygen consumption rate (OCR) and extracellular acidification rate (ECAR)

C28/I2 chondrocytes were plated at 10,000 cells per well in Seahorse XF96 Cell Culture Microplates (Agilent Technologies) and incubated overnight to achieve 70–80 % confluence. Growth medium was switched to XF Assay medium (Agilent) - a bicarbonate- and HEPES-free minimal media before starting the metabolic assays. The basal medium for mitochondrial respiration analysis contained 25 mM glucose and 4 mM L-glutamine but only 4 mM L-glutamine for the glycolytic flux. Plates incubated for 60 min at 37 °C in non- CO_2_ incubator prior to making Extracellular Flux. ECAR assessment: 10 mM Glucose, 2 μM Oligomycin, 100 mM 2-DG were subsequently added to the assay medium. For the OCR measurement, 2 μM oligomycin, 2 μM FCCP, and 1 μM of both rotenone and antimycin A were added one after another. Cellular energetics were followed in real time by Agilent Seahorse XF96 Extracellular Flux Analyzer. Resultant metabolic parameters were normalized relative to cell density and quintuplicate replicates were performed for each condition.

### 2.20 Catalase (CAT), superoxide dismutase (SOD), and malondialdehyde (MDA) analyses

Oxidative stress was determined by assessing the level of CAT, SOD and MDA using commercial assay kits (Beyotime, China). The CAT activity was measured by kinetic determination of H2O2 decomposition. SOD activity was measured according to its ability to inhibit the reduction of WST-8 by superoxide anions. MDA levels were determined by its reaction with thiobarbituric acid in the presence of acid, to form a chromogenic adduct. The standard curves were run for each marker and the results are presented as an arithmetic mean ± Standard deviation.

### 2.21 Protein microarray

Following the 28-d induction co-culture of MDVs and chondrocyte pellets, inflammatory markers in chondrocytes were detected using a protein microarray (AAH-BLG-TOL-4, RayBiotech Co., Ltd., China). The array includes antibodies targeting 181 proteins involved in the Toll/NOD-like receptor pathways. After binding cellular proteins to the primary antibodies on the custom array, nonspecifically bound proteins were removed by washing. For colorimetric detection, HRP-conjugated secondary antibodies specific to target proteins were added. followed by development with a chromogenic substrate. Chemiluminescent signal quantification established target protein presence and relative abundance. Biomarker expression levels were calibrated against HRP reference spots through grayscale densitometry analysis, with signal intensities normalized to these standardized controls. Differentially expressed proteins (DEPs) were identified and sorted using hierarchical clustering and visualized as heatmaps.

### 2.22 DNase treatment and Re-isolation of mitochondria-derived vesicles

To specifically assess the role of mtDNA in MDV-induced cGAS-STING activation, we treated isolated MDVs with DNase I (1 U/μg MDV protein; Sigma-Aldrich) in PBS containing 5 mM MgCl_2_ at 37 °C for 30 min to enzymatically degrade any external or loosely associated DNA, including mtDNA. Following the DNase treatment, 5 mM EDTA was added to stop the reaction, and the MDVs were re-isolated from the solution using ultracentrifugation (100,000×*g* for 70 min at 4 °C) to remove the DNase and digested DNA. Pellet MDVs were resuspended in PBS for the following experiment. This step made sure that the observed effects were caused by the DNase-sensitive DNA cargo inside MDVs, and not just because of a general stop of things from happening.

### 2.23 Intra-articular injection of MDVs in an OA rat model

Following relevant ethical requirements, the experiment protocols for animal subjects have been approved by the ethical committee of Guangzhou women and children's medical center. These investigations strictly adhered to the ARRIVE 2.0 reporting framework and complied with China's national standard GB/T 35892-2018 ″Guidelines for the Ethical Review of Laboratory Animal Welfare”. Twenty five 8-week-old male Sprague-Dawley rats were sourced from the Guangzhou Women and Children's Medical Center Laboratory Animal Facility. Animals were maintained under standardized conditions: ambient temperature 18–25 °C, relative humidity 40–70 %, 12 h light-dark cycles, with ad libitum access to food and water throughout the study duration. Bedding composed of corncobs and wood shavings was changed two to three times per week to ensure hygiene. OA was induced in the rats by intra-articular injection of 0.2 mL of monosodium iodoacetate (MIA; 5 g/mL, I2512, Sigma-Aldrich, USA) into the knee joints. Prior to MIA administration, rats were anesthetized via intraperitoneal injection of pentobarbital sodium (50 mg/kg). The OA model was validated via assessment of plantar pain thresholds four weeks post-induction.

### 2.24 Grouping of OA model rats

Randomization via a number table was employed to assign the rats to five distinct groups: Sham, OA, WT-MDV, OA-MDV, and OA-MDV with SN-011. Four weeks after the MIA injection, each group of experiments received 3 injections into the knee joint cavity of the intra-articular injection of different days, injected once every 3 days. The injected volume was 0.2 mL per dose, consisting of the following treatments: OA group-saline (vehicle control); WT-MDV group-WT MDVs (12 μg/mL); OA-MDV group-OA-derived MDVs (12 μg/mL); and SN-011 group-STING inhibitor SN-011 (30 mg/kg). There were no post injection problems like infection around the wound or having problems eating. The rats did not get much heavier or smaller. After 2 weeks since the last shot, the rats were killed with excess CO_2._ Harvested articular cartilage tissue by using a handheld chainsaw, and then fixed in 4 % PFA for evaluation.

### 2.25 X-ray and micro-computed tomography (Micro-CT) analysis

Radiography of the knee joint was employed to analyze the knee structure of all experimental groups'rats. First, X-rays were taken with the Faxitron MX-20 specimen radiography system to look at typical signs of osteoarthritis like joint space getting smaller and bony bumps called osteophytes forming.

Subsequently, high-resolution micro-CT scanning was carried out using a Scanco Medical μ-CT 35 imaging system with the following parameters: voltage 70 kV, current 114 μA, integration time 300 ms, and isotropic voxel size 10 μm. Three-dimensional reconstruction was performed on specific regions of interest: 1) the subchondral region of the tibial plateau was scanned, and the trabecular bone area starting 100 μm distal to the growth plate was selected for analysis of bone volume fraction (BV/TV) after excluding cortical bone; 2) three-dimensional morphological analysis of osteophytes at the margins of the femoral condyles and tibial plateau was conducted, with quantitative assessment of their relative volume. All quantitative analyses were based on n = 5 independent biological replicates per group, with data presented as mean ± standard deviation.

### 2.26 Hematoxylin and eosin (H&E) staining

Articular tissue sections from Sprague-Dawley rat knee joints were histologically processed using hematoxylin and eosin (H&E) staining kits (G1120, Solarbio, China). Quantitative assessment of Osteoarthritis progression severity using a modified Mankin scoring system, where 3 independent pathologists blinded to group assessment the degree of cartilage degeneration for each histological section. Final Mankin score is the median of three replicates for each sample. Mankin Scale is an evaluation of four variables; tissue architecture (0–6), cellular density (0–3), extracellular matrix dye affinity (0–4) and tidemark continuity (0–1) for a total of 14 possible points per specimen. The final histopathological grade given to each individual specimen was the highest-grade pathological alteration identified on tissue sections. For differences between assessor scores, the highest score is recorded.

### 2.27 Safranin O/Fast Green staining

Knee joint sections from rats were stained with Saffron O/Fast Green (G1531, Solarbio, China) to determine the extent of cartilage degeneration. Damage severity was graded according to OARSI using a validated 0–6 scale, focusing on degenerative changes in the medial tibiofemoral compartment. Five slices of articular cartilage tissue from each joint was scored, and the final score was made up of the highest score found and the total of the four highest scores.

### 2.28 Immunological staining

For characterizing the expression of the target protein, IF or IHC staining was carried out on knee joint sections. Tissue sections processed via standard procedures then blocked for 1 h in 0.5 % Sequestering Reagent (PerkinElmer, USA), following an overnight incubation with primary antibodies at room temperature, the samples were subsequently probed for 1 h with the appropriate fluorescently-conjugated secondary reagents. All antibody details are provided in Table S2. Staining results were visualized using either an inverted or confocal microscope and quantified using ImageJ. Specifically, images were split into individual fluorescent channels, converted to an 8-bit format, and analyzed by measuring grayscale intensity after applying appropriate thresholds.

### 2.29 Statistical analysis

Data represent mean ± SD from ≥3 independent experiments. Statistical comparisons employed Student's t-test (two groups) or one-way ANOVA (>2 groups) using GraphPad Prism v10.0. Significance thresholds: ∗*p* < 0.05, ∗∗*p* < 0.01, and ∗∗∗*p* < 0.001. Differences labeled “ns” were not considered statistically significant.

## 3 Results

### 3.1 MDVs are significantly increased in the synovial fluid of patients with OA

**Fig. 1:**
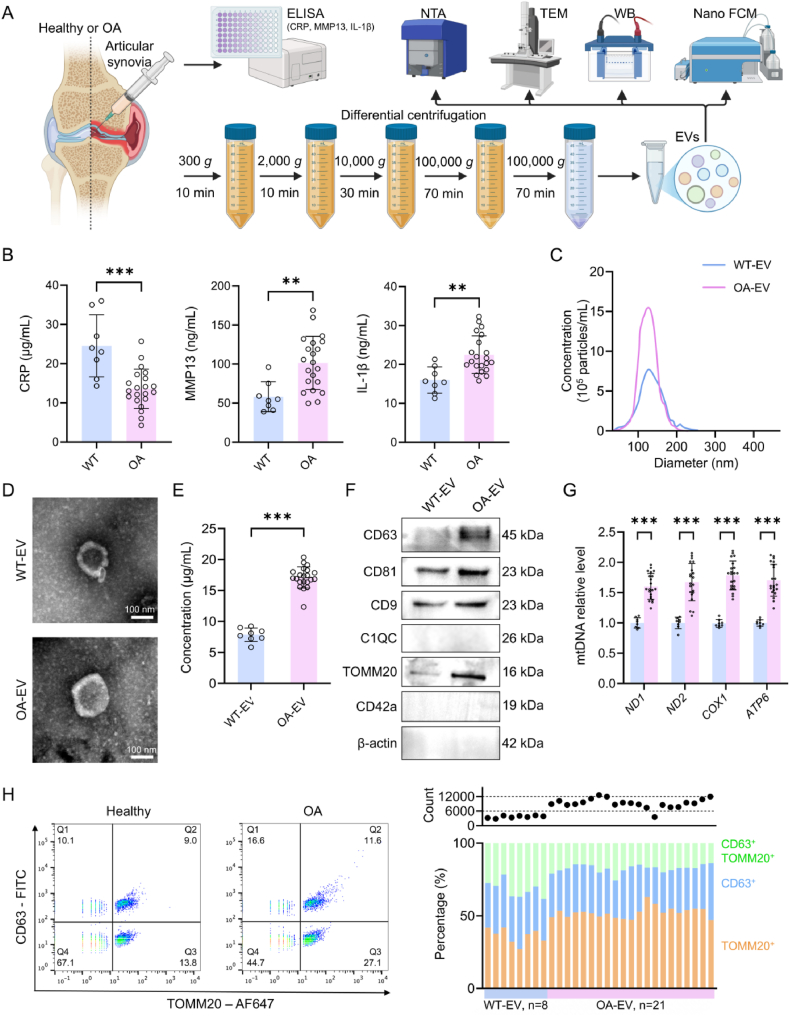
Isolation and characterization of extracellular vesicles (EVs) from the synovial fluid of patients with osteoarthritis (OA). (A) Schematic overview of the experimental overflow. (B) Quantification of C-reactive protein (CRP), matrix metalloproteinase 13 (MMP13), and interleukin-1β (IL-1β) using ELISA (WT-EVs, n = 8. OA-EVs, n = 21). (C) Particle size distribution of EVs measured using nanoparticle tracking analysis (NTA). (D) Transmission electron microscopy (TEM) images showing EV morphology. (E) Protein concentration of EVs measured using the bicinchoninic acid (BCA) assay (WT-EVs, n = 8. OA-EVs, n = 21). (F) WB analysis of EV-associated biomarkers. (G) MtDNA content in EVs (WT-EVs, n = 8. OA-EVs, n = 21). (H) NanoFCM analysis of EV subpopulations in synovial fluid from healthy individuals and patients with OA (WT-EVs, n = 8. OA-EVs, n = 21). ∗*p* < 0.05, ∗∗*p* < 0.01, ∗∗∗*p* < 0.001, and ∗∗∗∗*p* < 0.0001.

Considering that inflammation plays a key part in OA progression and EVs are becoming crucial for intercellular communication, we looked at EV subpopulations and inflammatory markers in synovial fluid from OA patients. First, we measured the three OA-related inflammatory factors, CRP, MMP13, and IL-β, in the OA group of patients and in the WT group of patients with trauma but no OA. EVs were then isolated from synovial fluid by differential centrifugation (Fig. 1A). Compared to WT samples, OA samples exhibited higher expression of MMP13 (101.50 ± 67.43 ng/mL, 174.49 % increase) and IL-β (22.49 ± 6.69 ng/mL, 140.47 % increase). Conversely, the CRP levels were notably elevated in the WT samples (Fig. 1B), which is in keeping with it being an acute-phase injury marker. These results match the expected inflammatory profile of the study group.

Subsequently, we examined the EVs present in the synovial fluid of both groups [31]. By using NTA, we found out that the particles of both groups were mainly distributed between 80 and 200 nm. In term of the number of particles OA-EVs reached up to 10^6^ particles/mL, this number is far above the level that is reached for WT-EVs (Fig. 1C). TEM revealed typical vesicular morphology, with sizes of 121.45 ± 24.58 nm and 138.29 ± 16.70 nm, respectively (Fig. 1D). Given the different EV subpopulations, we performed WB analysis to detect subtype-specific biomarkers. Prior to this, protein quantification by BCA assay (Fig. 1E) confirmed that OA-EV samples had more than twice the protein concentration than that of WT-EVs-consistent with the NTA findings. This increase was more pronounced than that observed for EXO markers, suggesting that MDVs represent the most substantially elevated EV subtype in the synovial fluid of patients with OA.

WB (Fig. 1F) confirmed the expression of canonical EV markers CD63, CD81, and CD9-shared by both EXOs and MVs [32]. These markers were expressed in both WT-EVs and OA-EVs, with higher levels in OA-EVs, indicating an increase in EXOs in the synovial fluid of patients with OA. While apoptotic bodies can also be smaller than 200 nm, we did not detect their marker, C1QC, in the EV samples. Similarly, CD42a, a marker for platelet-derived vesicles, was also undetectable. TOMM20, a core subunit of the mitochondrial TOM complex that mediates protein import, was significantly upregulated in OA-EVs and serves as a marker of MDVs [33]. The findings support that MDVs is a significantly increased EV subpopulation in the synovial fluid of OA patients, but the source cell of MDVs and their exact roles in OA development are still unknown.

To support the presence of MDV in synovial fluid, we assessed the presence of mtDNA within EVs (Fig. 1G). By comparing the relative amounts of mtDNA *ND1*, *ND2*, *COX1* and *ATP6*, it can be seen that OA-EVs contain more mtDNA as expected, *p* < 0.001. In order to establish the relationship between the amount of MDV biomarker TOMM20 and mitochondrial mtDNA content, ELISA is used to detect the amount of TOMM20 protein. The TOMM20 level in WT-EV was significantly lower than that in OA-EV (118.01 ± 41.00 pg/mL vs. 571.19 ± 143.92 pg/mL), consistent with the results from WB. Linear regression analysis shows that there exists a low correlation between TOMM20 and mtDNA in the WT-EVs, but a high positive correlation is observed between TOMM20 and mtDNA in the OA-EVs, as shown in Fig. S2A. At the same time, TFAM, as a mtDNA binding protein, also showed that its protein levels were closely correlated with the mtDNA content (Fig. S2B). Thus, TOMM20+ OA-EVs carried more mtDNA.

NanoFCM analysis showed different synovial fluid EV profiles in WT and OA groups (Fig. 1H). CD63 was used as an exo/mvs biomarker and TOMM20 was used as an mdv biomarker. WT samples contained a total EV concentration of 3545.33 ± 494.61 particles/3 mL, with TOMM20+ EVs making up 36.36 ± 5.82 % of the population. In contrast, the OA samples had significantly higher EV concentrations (9566.00 ± 994.67 particles/3 mL; 2.7-fold) and an increase in TOMM20+ EVs (51.44 ± 1.90 %). This quantitative change shows more MDV secretion (TOMM20^+^ EVs) and greater EXO/MVs (CD63^+^ EVs) in OA disease. Together, the data point to disease-associated EV subpopulation remodeling, which includes both quantitative and qualitative changes to surface proteins.

### 3.2 Single-cell sequencing reveals consistent trends between VSP35 expression and MDV abundance in OA

**Fig. 2:**
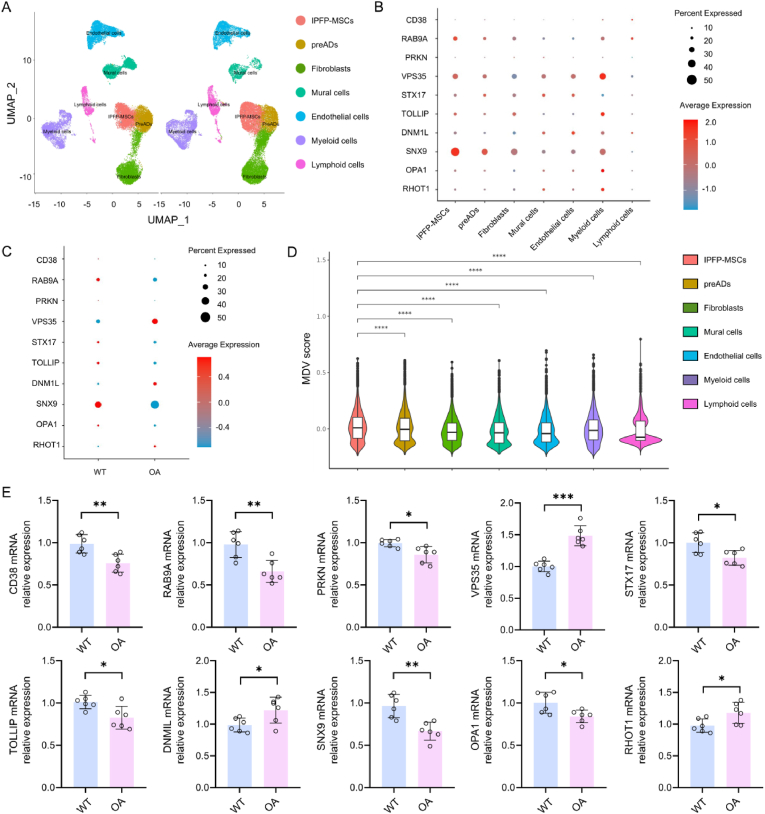
Single-cell sequencing of wild-type (WT) and OA infrapatellar fat pad mesenchymal stem cells (IPFP-MSCs). (A) Split-UMAP showing cellular composition in WT and OA groups. (B) Expression profiles of genes associated with mitochondria-derived vesicle (MDV) biogenesis and secretion across cell types. (C) Comparative analysis of MDVs formation related genes expression in WT versus OA groups. (D) Score of MDVs formation related genes in OA group. (E) qPCR validation of gene expression in WT and OA joint tissues. ∗*p* < 0.05, ∗∗*p* < 0.01, ∗∗∗*p* < 0.001, and ∗∗∗∗*p* < 0.001.

Analysis was done using Ding's and Qin's GSE216651 single-cell sequencing data [2], on the transcriptomic landscape of WT and OA groups. Split-UMAP showed different cell clusters (Fig. 2A), and subpopulation identities were confirmed by marker genes (Fig. S3A). Checking out MDV biogenesis-related transcripts (*CD38*, *RAB9A*, *PRKN*, *VPS35*, *STX17*, *TOLLIP*, *DNM1L*, *SNX9*, *OPA1*, *RHOT1*) and observing the pattern of gene transcripts amongst all cells (Fig. 2B), spatial enrichment of these genes was found in OA-IPFP-MSCs (Fig. S3B), indicating that these cells may be specialized to produce MDV. While *RAB9A* and *SNX9* expression was increased in the WT samples, *VPS35* was increased in an OA-specific manner in IPFP-MSCs (Fig. 2C). Based on the highly expressed *VPS35* in IPFP-MSCs and the highest expression in the OA group, we assumed that it was closely related to the MDV secretion and was confirmed through the UMAP of OA samples (Fig. 2D). Module scoring analysis showed that OA-induced IPFP-MSCs have a much higher activation of MDV-related genes than that of the other stromal cell (Fig. S4). Moreover, qPCR analysis on collected joint tissues (Fig. 2E) also showed gene expression profiles consistent with the single-cell sequencing data. In our validation, certain genes-such as *CD38* and *PRKN*-exhibited markedly more pronounced differential expression. Taken together, these findings indicate that the increased MDV abundance in OA synovial fluid may originate from IPFP-MSCs and could be regulated by *VPS35*. However, further investigation is necessary to clarify the role of MDVs in OA pathogenesis.

### 3.3 Isolation and characterization of MDVs derived from IPFP-MSCs

**Fig. 3:**
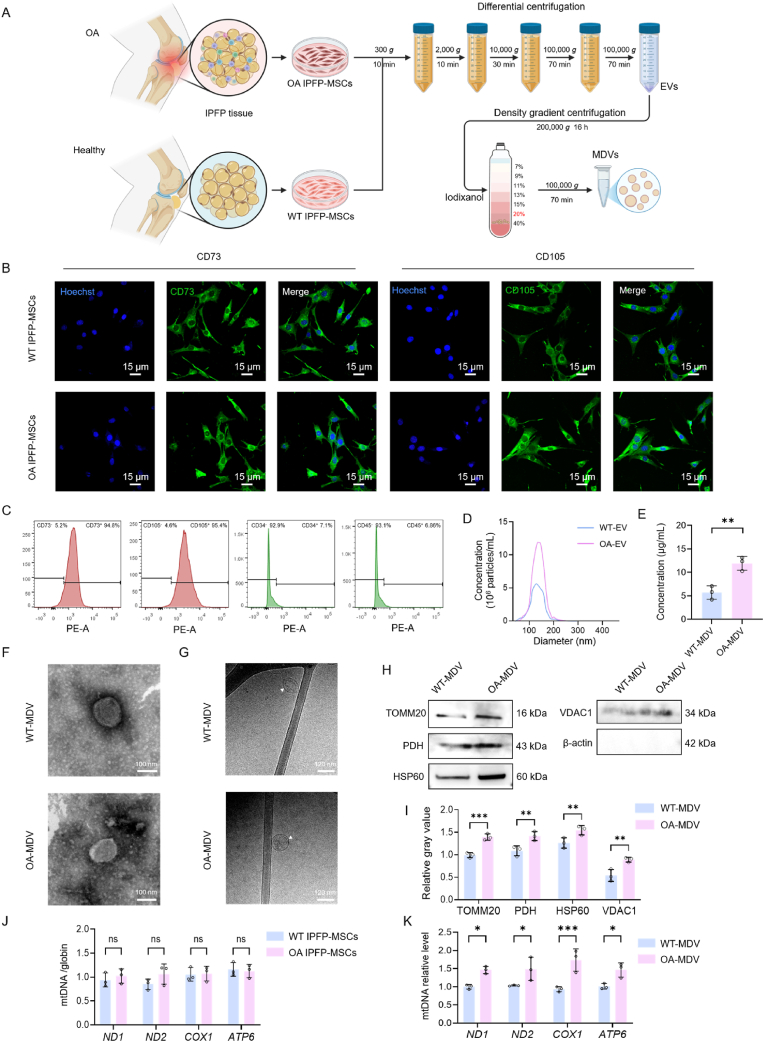
Isolation and characterization of MDVs derived from IPFP-MSCs. (A) Schematic representation of IPFP-MSC isolation from human knee joint tissues and subsequent MDV purification. (B) IF staining of IPFP-MSC surface markers. (C) Flow cytometric analysis of IPFP-MSC surface markers. (D) Particle size distribution of EVs as measured using NTA. (E) Protein concentration of MDVs quantified using the BCA assay (n = 3). (F) TEM images showing MDV morphology. (G) cryo-TEM images showing MDV morphology. (H) WB analysis of MDV-associated biomarkers. (I) Quantitative analysis of MDV-associated biomarkers by WB (n = 3). (J) Relative mtDNA content in IPFP-MSCs (n = 3). (K) Relative mtDNA content in MDVs (n = 3). ∗*p* < 0.05, ∗∗*p* < 0.01, and ∗∗∗*p* < 0.001.

IPFP tissue was obtained during joint replacement in patients with OA and during surgery in patients with trauma. We extracted normal WT IPFP-MSCs and inflammatory OA IPFP-MSCs from the two groups. To investigate whether the observed increase in MDVs in OA synovial fluid originates from IPFP-MSCs, we isolated MDVs via density gradient centrifugation. (Fig. 3A). The cultured cells were subsequently examined using IF and flow cytometry, which confirmed high expression of stem cell markers (CD105 and CD73) and absence of hematopoietic or leukocyte lineage markers (CD34 and CD45) (Fig. 3B and C). Following differential and density gradient centrifugation, we successfully isolated MDVs. In NTA analysis (Fig. 3D), both WT-MDVs and OA-MDVs had sizes between 100 and 200 nm, but the OA-MDVs concentration was about 1 × 10^7^ particles/mL, which was about two times of the concentration of WT-MDVs. The reason for this was that OA-MDVs were isolated from a large volume of cell culture medium rather than synovial fluid. Similarly, the protein level in OA-MDVs was significantly higher than in WT-MDVs, with a 204.73 % increase compared to WT-MDVs (12.98 ± 2.42 μg/mL, p < 0.01) (Fig. 3E). TEM and cryo-TEM showed that both WT-MDVs and OA-MDVs presented with sphere-shaped, bilayer membrane structures (Fig. 3F and G). WB and quantitative analysis (Fig. 3H and I) were carried out to identify the MDV biomarkers and their mitochondrial components. TOMM20, PDH, and HSP60 were all found in both groups of MDVs. These are proteins that sit in the mitochondrial outer membrane, inner membrane, and matrix, respectively. Mitochondrial housekeeping protein VDAC1 was found in MDVs, but cytoplasmic housekeeping protein β-actin was not. To determine if the difference in mtDNA content in MDVs results from a change in cellular mtDNA production or selective packaging of mtDNA during vesicle biogenesis, we measured mtDNA levels in both IPFP-MSCs and MDVs isolated from WT and OA conditions. Cellular mtDNA were quantified with *ND1/β-globin*, *ND2/β-globin*, *COX1/β-globin*, and *ATP6/β-globin* ratios, which showed no statistical difference between WT-IPFP-MSCs and OA-IPFP-MSCs (Fig. 3J). On the other hand, analysis of mtDNA in isolated MDVs shows high relative Cts of *ND1*, *ND2*, *COX1* and *ATP6* for OA MDVs when compared to WT MDVs (Fig. 3k). These results suggest that the increased mtDNA in OA-MDVs is likely due to enhanced packaging during MDV biogenesis rather than elevated cellular mtDNA production.

Collectively, our *in vitro* experiments successfully isolated IPFP-MSCs and their derived MDVs. Similar to EVs in synovial fluid EVs, MDVs were elevated in the OA IPFP-MSC group. Although both WT-MDVs and OA-MDVs expressed characteristic mitochondrial and MDV proteins, OA-MDVs contained more mtDNA, which suggests that OA-MDVs may contribute to activation of DNA-sensing pathways in OA target cells.

### 3.4 MDVs contain differentiated mitochondrial protein components

**Fig. 4:**
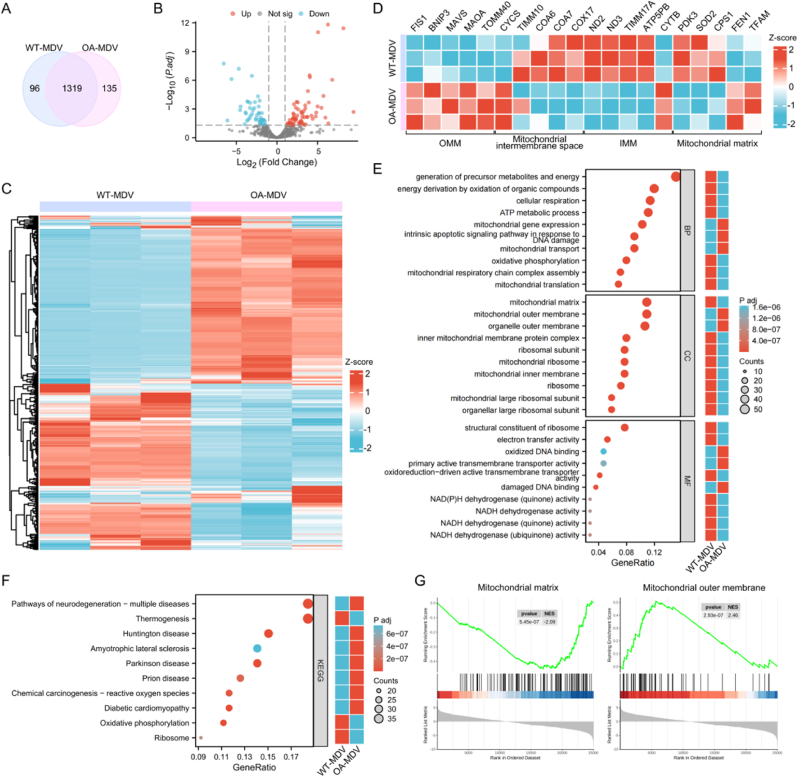
Proteomic profiling reveals distinct protein composition and functional characteristics in MDVs. (A) Venn diagram illustrating the numbers of proteins identified uniquely in WT-MDVs (96), uniquely in OA-MDVs (135), and shared between both groups (1319). (B) Volcano plot of 2-fold DEPs. (C) Heatmap of 2-fold DEPs. (D) Heatmap of top 5 DEPs in different mitochondrial substructures. (E) GO enrichment analysis. (F) Enrichment analysis of KEGG signaling pathway. (G) GSEA diagram of mitochondrial matrix and outer mitochondrial membrane.

To characterize the protein composition of MDVs, we performed proteomic profiling of WT-MDVs and OA-MDVs. Among the 1550 proteins identified, 1319 were commonly expressed in both types of MDVs, while 96 and 135 proteins were uniquely detected in WT-MDVs and OA-MDVs, respectively (Fig. 4A). A total of 376 DEPs with a 2-fold change or greater were identified (Fig. 4B and C). Based on the subcellular localization of these DEPs, we generated heatmaps for highly divergent proteins associated with the mitochondrial outer membrane (OMM), intermembrane space, inner membrane (IMM), and matrix. Overall, the heatmap showed that OA-MDVs were primarily enriched in OMM components, such as the TOMM40, whereas WT-MDVs exhibited a broader representation of other mitochondrial structural components. Specifically, WT-MDVs included many subunits of electron transport chain complexes, such as COA6, COA7, COX17, ND2, ND3, and ATP5B, and also proteins from the MRPL ribosome family. In contrast, OA-MDVs were enriched in OMM proteins such as BNIP3 (involved in apoptosis) and MAVS (involved in innate immune response) and IMM protein CYCS involved in apoptosis initiation. OA-MDVs were also enriched for the mtDNA binding protein TFAM. Based on our previous observation of a positive relationship between TFAM and mtDNA, the increase in amount of mtDNA seen in OA-MDVs over WT -MDVs is now supported, another protein which is higher in OA- MDV's versus WT -MDVs is FEN1, mitochondrial matrix protein which plays role in the excision repair of mtDNA that has experienced oxidative damage (Fig. 4D). These findings suggest that WT-MDVs and OA-MDVs have different protein expression profiles with WT-MDVs containing mainly mitochondrial respiratory chain proteins and OA-MDVs mainly containing OMM and mtDNA proteins.

Based on the DEPs, GO enrichment analysis revealed distinct functional characteristics between WT-MDVs and OA-MDVs. WT-MDVs were significantly enriched in biological processes such as generation of precursor metabolites and energy, energy derivation by oxidation of organic compounds, cellular respiration, ATP metabolic process, and oxidative phosphorylation. Cellular component terms strongly associated with WT-MDVs included the mitochondrial inner membrane, mitochondrial respiratory chain complex, mitochondrial large ribosomal subunit, and organellar large ribosomal subunit. Molecular functions enriched in WT-MDVs featured structural constituent of ribosome, electron transfer activity, NAD(P)H dehydrogenase (quinone) activity, and primary active transmembrane transporter activity. OA-MDVs showed prominent enrichment in biological processes including mitochondrial transport, intrinsic apoptotic signaling pathway in response to DNA damage, and mitochondrial gene expression. Cellular components overrepresented in OA-MDVs comprised the mitochondrial outer membrane, organelle outer membrane, and mitochondrial matrix. Molecular functions specifically associated with OA-MDVs included oxidized DNA binding, damaged DNA binding, and oxidoreduction-driven active transmembrane transporter activity (Fig. 4E). KEGG pathway analysis further demonstrated that WT-MDVs were predominantly enriched in oxidative phosphorylation, thermogenesis, and ribosome pathways. OA-MDVs were notably associated with pathways of neurodegeneration-including Huntington disease, amyotrophic lateral sclerosis, Parkinson disease, and prion disease-as well as diabetic cardiomyopathy and chemical carcinogenesis involving reactive oxygen species (Fig. 4F). Gene Set Enrichment Analysis (GSEA) verified these functional patterns and confirmed that the two MDV types had different types of proteins in their mitochondria structures (Fig. 4G). The stark contrast here suggests that we have a kind of stimulus-specific packaging system in which the Mitochondrial Disease Mutant (MDM) from normal (healthy) mitochondria are mainly loaded with a set of the metabolism machinery found in the matrix whereas those from stressed ones tend to be packed more with outer membrane proteins related to signal or quality control, which may decide their different fates.

The results revealed, at the proteomic level, that the MTs of WT-MDVs and OA-MDVs have different protein profiles, indicating that the two kinds of MDVs might have different effects on the target cells.

### 3.5 MDVs inhibit chondrogenic differentiation *in vitro*

**Fig. 5:**
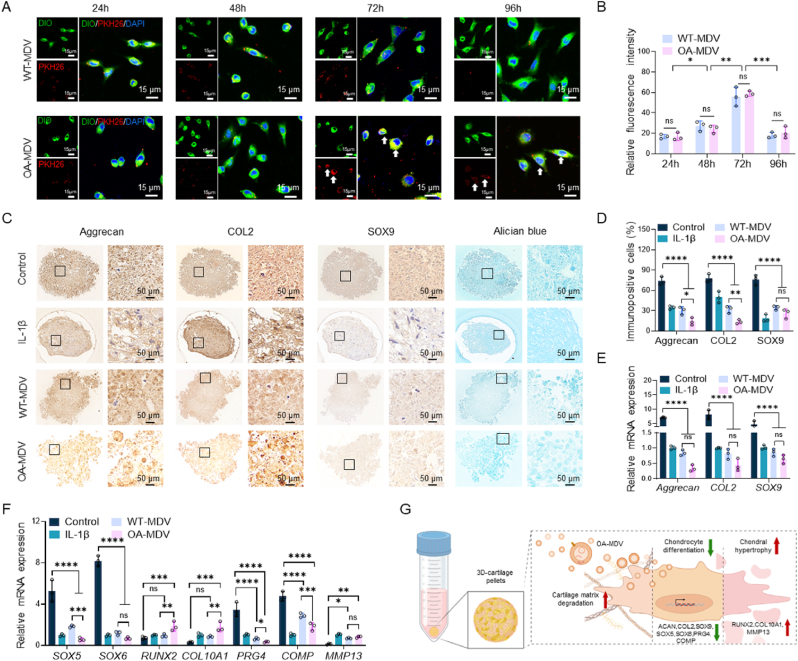
MDVs inhibits chondrogenic differentiation *in vitro*. (A) Fluorescence images of PKH26-labeled MDV (red) uptake by C28/I2 cells (cell membrane is labeled with DIO, green. The nucleus is labeled with DAPI, blue) at 24, 48, 72, and 96 h. (B) Quantitative analysis of fluorescent intensity of internalized MDVs. (n = 3) (C) Immunohistochemical (IHC) staining of aggrecan, COL2, and SOX9, along with Alcian Blue staining of chondrocyte pellets after 21 d of co-culture with MDVs. Magnification: 50 × (left) and 200 × (right). (D) Proportion of positive IHC-stained cells (n = 3). (E) mRNA expression levels of *aggrecan*, *COL2*, and *SOX9* (n = 3). (F) mRNA expression levels of *SOX5*, *SOX6*, *RUNX2, COL10A1*, *PRG4*, *COMP* and *MMP13*. (n = 3). (G) Schematic representation of the 3D chondrosphere co-culture with MDVs. ∗*p* < 0.05, ∗∗*p* < 0.01, ∗∗∗*p* < 0.001 and ∗∗∗∗*p* < 0.0001.

The effect of MDVs derived from IPFP-MSCs on OA was investigated in C28/I2 chondrocytes *in vitro*. First, an internalization assay was conducted to determine whether IPFP-MSC-derived MDVs could be taken up by chondrocytes (Fig. 5A). Internalization of both MDV types peaked at 72 h (Fig. 5B), and fluorescence imaging revealed dot-shaped PKH26-labeled MDVs within the cytoplasm of chondrocytes. However, after 72 and 96 h of co-culture with OA-MDVs, PKH26 fluorescence appeared along the cell membrane, possibly indicating direct fusion of OA-MDVs with the target cells, as indicated by white arrows.

To further clarify the pathways of MDV uptake, we used endocytosis and membrane fusion inhibitors to study the internalization of WT-MDV and OA-MDV (Fig. S5A). Fluorescence intensity quantification showed that inhibiting clathrin-mediated endocytosis (with Dynasore), macropinocytosis (with Cytochalasin D), phagocytosis (with Amiloride), and membrane fusion (with Z-Phe-Phe-Phe-OH) reduced WT-MDV internalization efficiency to different extents. Cytochalasin D reduced internalization efficiency to 46.33 ± 4.50 %, which was less than half of the untreated control. In contrast, OA-MDV internalization remained unchanged when clathrin-mediated endocytosis and macropinocytosis were inhibited (*p* > 0.05). However, in the presence of phagocytosis and membrane fusion inhibitors, OA-MDV internalization efficiency dropped significantly to 28.67 ± 3.09 % and 23.00 ± 3.45 % of the untreated control, respectively (Fig. S5B and C). Compared to WT-MDV, OA-MDV relies more on phagocytosis and membrane fusion for internalization, potentially leading to differential functional outcomes.

To further explore the biological role of MDVs in OA, both groups of MDVs were co-cultured with chondrocyte pellets *in vitro*. IHC staining demonstrated that the expression of aggrecan and COL2 in the chondrocyte pellets from the OA-MDV group was the lowest among all groups and even lower than that from the IL-1β group. In contrast, SOX9 expression was the lowest in the IL-1β group. However, SOX9 expression in the OA-MDV group was found to be lower than all groups except IL-1β. Alcian blue staining revealed that chondrocytes in the chondrocyte pellets co-cultured with MDVs exhibited reduced proliferation and smaller cell volume, with the most pronounced difference observed between the OA-MDV and Control groups (Fig. 5C and D). Based on the qPCR results (Fig. 5E and F), IL-1β and OA-MDV induce down regulation of cartilage matrix genes (*ACAN*, *COL2A1*, *COMP*, *PRG4*) and key chondrogenic transcription factors (*SOX9*, *SOX5*, *SOX6*), while concurrently upregulating hypertrophic markers (*RUNX2*, *COL10A1*) and degradative enzymes (*MMP13*), compared to normal chondrocytes. The 3D chondrosphere model revealed MDV-induced structural disintegration, characterized by pericellular protease activity and abnormal collagen deposition, mechanistically linking mitochondrial stress to inflammatory matrix catabolism (Fig. 5G). These findings suggest that IPFP-MSC-derived MDVs can be internalized by chondrocytes, with OA-MDVs partially acting on target cells through direct membrane fusion. This mechanism may contribute to accelerated chondrocyte degradation and OA progression.

### 3.6 MDVs-induced oxidative stress and mitochondrial dysfunction

**Fig. 6:**
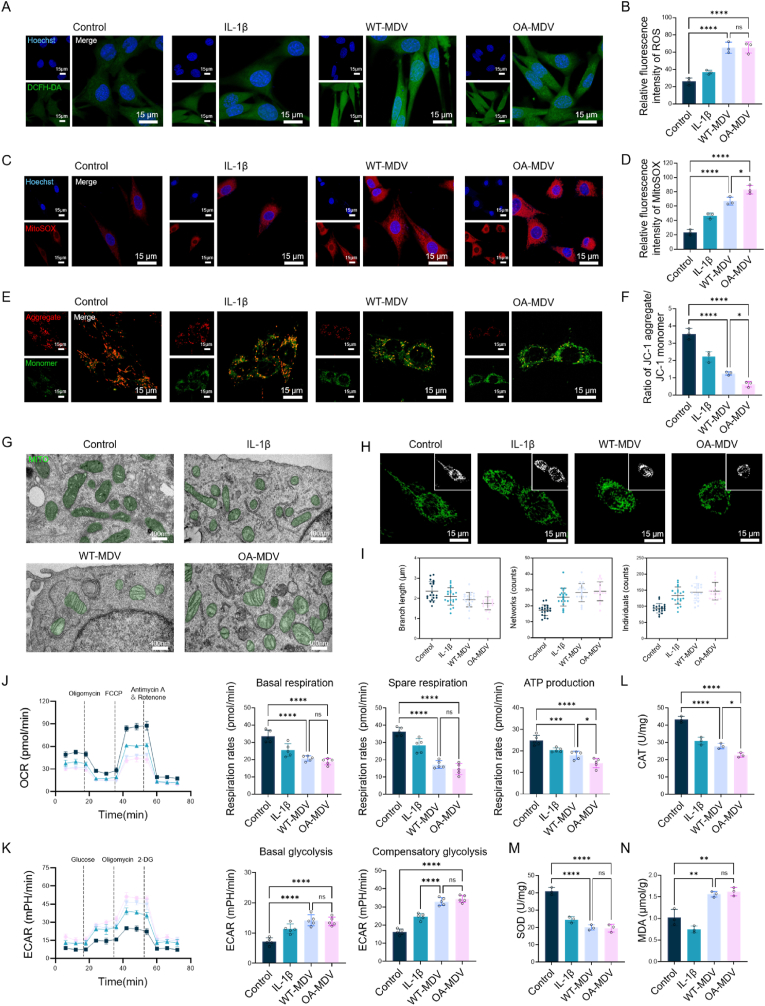
MDVs exacerbate oxidative stress and impair mitochondrial function. (A) Fluorescence microscopy images of intracellular ROS. (B) Quantitative analysis of ROS fluorescence intensity (n = 3). (C) Fluorescence microscopy images of MitoSOX. (D) Quantitative analysis of MitoSOX fluorescence intensity (n = 3). (E) JC-1 staining images showing mitochondrial membrane potential. (F) Quantification of the JC-1 aggregate-to-monomer fluorescence ratio (n = 3). (G) TEM Analysis of mitochondria (green) in chondrocytes. (H) Representative confocal images of mitochondrial morphology. (I) Quantitative analysis of mitochondrial branch length, network complexity, and counts (n = 3). (J) Seahorse analysis of mitochondrial oxygen consumption rate (n = 5). (K) Extracellular acidification rate reflecting glycolytic activity (n = 5). Quantification of (L) catalase activity (n = 3), (M) superoxide dismutase activity (n = 3), and (N) malondialdehyde levels (n = 3). ∗*p* < 0.05, ∗∗*p* < 0.01, ∗∗∗*p* < 0.001, and ∗∗∗∗*p* < 0.0001.

To investigate the impact of MDVs on oxidative stress, the amount of ROS was examined throughout the groups (Fig. 6A and B). ROS in the IL-1β group is more than that in the control group, but both MDV groups show comparable and higher ROS levels compared with all other groups. MitoSOX red fluorescence staining revealed that compared to the control group, the IL-1β,WT-MDV, and OA-MDV treatments all increased mitochondrial ROS levels. The OA-MDV group had the strongest fluorescence, which meant that it promoted mitochondrial oxidative stress the best (Fig. 6C). The quantitative analysis (Fig. 6D) showed a significant increase of ROS after OA-MDV treatment. Then, we performed mitochondrial membrane potential test with JC-1 fluorochrome. IL-1β group showed lower membrane potential than control. MDV groups showed disrupted membrane potential that was lower than the other groups and the OA-MDV group displayed the largest reduction (Fig. 6E and F). TEM imaging shows great ultrastructural changes including mitochondrial swelling and damage in the IL-1β and OA-MDV treated chondrocytes which indicates much more severe inflammatory injury and cell stress compared with the control and MDV groups (Fig. 6G). In addition, we studied the morphology of mitochondria of every group to assess the changes brought about by MDV. The two MDV groups had smaller branch lengths and more fragmented and interconnected mitochondrial networks than the rest of the groups (Fig. 6H and I). These results indicate that MDVs can negatively affect the mitochondrial function and OA-MDVs have a stronger negative effect on mitochondria. In terms of dynamic mitochondrial regulation, all three groups including IL-1β, WT-MDV and OA-MDV increased the formation of fragmented mitochondrial network and isolated mitochondria, indicating mitochondrial stress.

In Seahorse analysis, MDV treatment greatly inhibited mitochondrial respiratory chain activity, as shown by a reduction in basal respiration, spare respiratory capacity, and ATP production, with OA-MDVs showing the worst effects (Fig. 6J). As with mitochondrial dysfunction, ECAR assays also showed a compensatory increase in glycolytic activity in the MDV treated groups, indicating a metabolic switch towards glycolysis to compensate for the mitochondrial energy deficit (Fig. 6K). CAT activity was significantly reduced in both MDV groups as compared to IL-1β and Control and reached the lowest level in OA-MDVs (Fig. 6L). At the same time, oxidative stress indicators (SOD, MDA) indicated reduced antioxidant activity and increased lipid peroxidation in MDV-treated cells (Fig. 6M and N). Together, these findings suggest that MDVs increase oxidative stress and mitochondrial homeostasis disruption via structural and functional damage. OA-MDVs were equal to the WT-MDVs at the same concentrations, but the fact that there is more OA-MDV indicates a more dangerous situation.

### 3.7 MDVs activate the cGAS-STING pathway, leading to inflammation

**Fig. 7:**
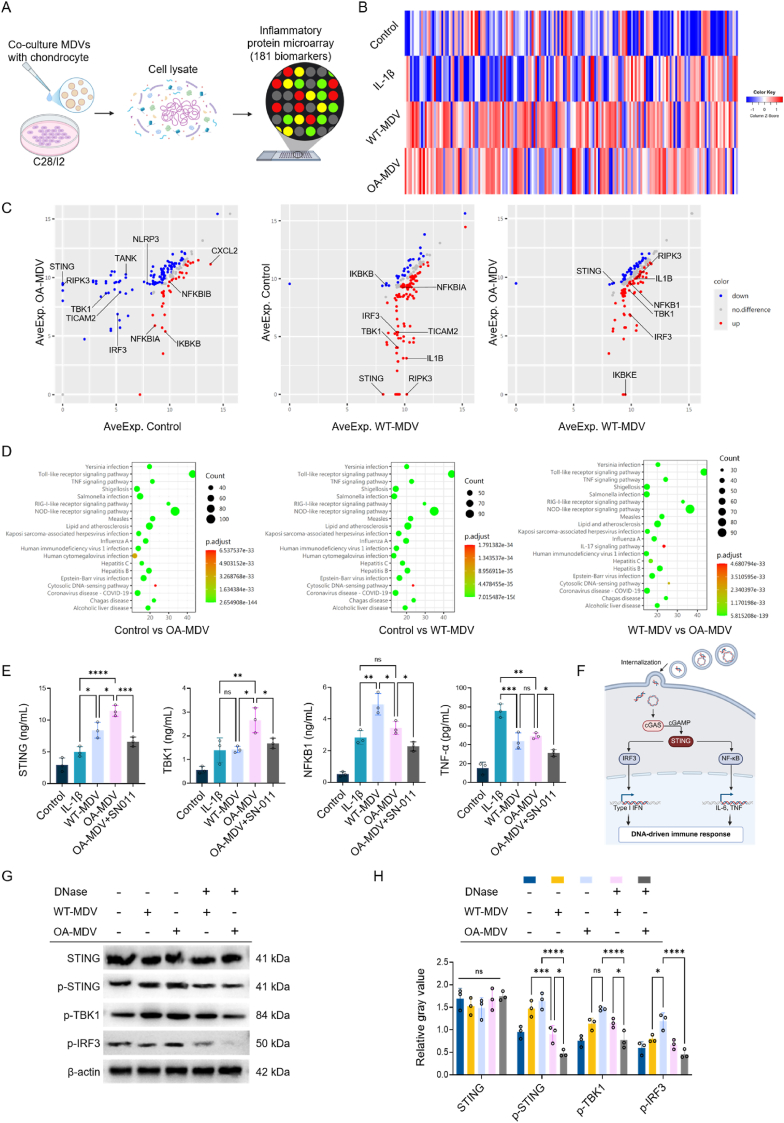
MDVs activate the cGAS-STING pathway, leading to inflammation. (A) Schematic of the protein microarray workflow. (B) DEPs with fold change in expression >2. (C) Scatter plots of DEPs. (D) KEGG pathway enrichment analysis of DEPs. (E) Validation of selected DEPs using ELISA (n = 3). (F) Schematic illustration of MDV-mediated activation of the cGAS-STING signaling pathway. (G) WB show STING pathway activation by OA-MDVs and its suppression by DNase pretreatment. (H) Quantitative analysis cGAS-STING pathway activation (n = 3). ∗*p* < 0.05, ∗∗*p* < 0.01, ∗∗∗*p* < 0.001, and ∗∗∗∗*p* < 0.0001.

To investigate the mechanism of action of MDVs, protein microarrays were used to analyze the expression of 181 protein components related to inflammatory pathways in chondrocytes co-cultured with MDVs (Fig. 7A and Fig. S6). Differential analysis of cellular protein expression was performed between OA-MDV vs. WT-MDV and IL-1β vs. Control groups. MDV groups both showed more expression of inflammation-related proteins than IL-1β and Control groups (Fig. 7B). Scatter plot showed that compared with the Control group, in pairwise comparison, the OA-MDV group mainly expressed inflammatory pathway-related proteins such as STING, RIPK3, TANK, IRF3, TBK1. WT-MDV group had the highest expression of RIPK3, TBK1, NFKB1, IL-1β, and other inflammatory pathway related protein. In terms of STING expression, OA-MDV group was slightly higher than that of WT-MDV group, and expression of the other inflammatory proteins were similar to WT-MDV group (Fig. 7C). Subsequently, KEGG pathway enrichment was carried out to learn about which pathways were involved. The pairwise comparisons of DEPs showed that there were significant differences in the cytosolic DNA-sensing pathway in all groups (Fig. 7D). This means DEPs are linked to the cGAS-STING pathway and that MDVs cause this pathway to work in chondrocytes, making the inflammation last and damaging the chondrocytes. We quantified the above hypothesis using ELISA to measure DEPs. Results showed that STING expression was elevated in both OA-MDV and WT-MDV, with a higher increase in OA-MDV. In order to find some differences between WT-MDV and OA-MDV, I explored these four factors on the cGAS-STING Pathway, STING, TBK1, NFKB1, TNF-ɑ. All 4 proteins displayed significantly higher expression levels in both MDV groups. Compared with WT-MDV, OA-MDV exhibited higher expression levels of STING, TBK1, and TNF-α by 136.62 % (11.79 ± 1.23 ng/mL), 137.5 % (2.42 ± 0.54 ng/mL), and 113.45 % (17.39 ± 1.38 pg/mL), respectively. However, NFKB1 expression was lower in the OA-MDV group than in the WT-MDV, at 73.56 % (4.58 ± 0.28 ng/mL) (Fig. 7E). In particular, SN-011 notably inhibited the upregulation of STING, TBK1 and TNF-α induced by OA-MDV and restored NFKB1 expression to that of the IL-1β group. These results support our hypothesis that MDVs act on chondrocytes through the cGAS-STING pathway. Schematic diagram showing how MDVs could activate this pathway (Fig. 7F). The WB analysis with Grayscale quantitative was performed and indicated strong activation of the STING pathway with upregulation of the Phospho-STING, Phospho-TBK1, Phospho-IRF3 (Fig. 7G and H). Critically, this activation was abolished when MDVs were pretreated with DNase I to degrade mtDNA, followed by ultracentrifugation to remove residual DNase/DNA fragments. This specificity control verified that the effect was attributable to MDV-carried mtDNA rather than non-vesicular components. The results align with our proteomic data showing enriched mtDNA-binding proteins (e.g., TFAM) in OA-MDVs and underscore mtDNA as the primary trigger of cGAS-STING signaling in chondrocytes. These findings provide direct evidence linking MDV cargo to innate immune activation in osteoarthritis pathogenesis.

### 3.8 Pro-inflammatory effect of MDVs through the cGAS-STING pathway *in vivo*

**Fig. 8:**
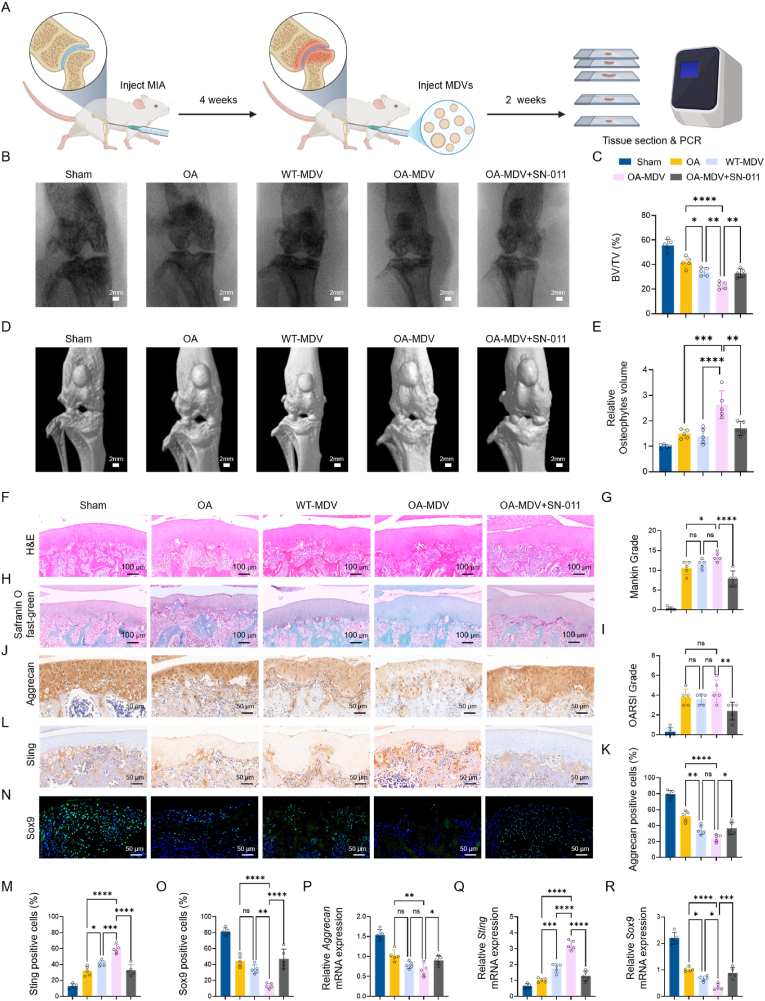
Effects of MDVs on OA pathogenesis *in vivo*. (A) Schematic illustration of the monosodium iodoacetate-induced OA mouse model. (B) Representative X-ray images of mouse knee joints. (C) Quantitative analysis of bone volume fraction across experimental groups. (D) Three-dimensional micro-CT reconstructions of knee joints. (E) Relative osteophyte volume quantification. (F) Hematoxylin and eosin staining of articular cartilage tissue at 2 weeks. (G) Markin score quantification (n = 5). (H) Safranin O/Fast Green staining of articular cartilage tissue at 2 weeks; (I) Osteoarthritis Research Society International scoring of cartilage damage (n = 5). (J) IHC staining of aggrecan in articular cartilage. (K) Quantification of aggrecan-positive cells (n = 5). (L) IHC staining of Sting in articular cartilage. (N) Quantification of Sting-positive cells (n = 5). (O) IF staining of Sox9 in articular cartilage. (P) Quantification of Sox9-positive cells (n = 5). (P–R) qPCR analysis of *aggrecan*, *Sting*, and *Sox9* mRNA levels (n = 5). ∗*p* < 0.05, ∗∗*p* < 0.01, ∗∗∗*p* < 0.001, and ∗∗∗∗*p* < 0.0001.

We investigated the biological function of MDVs through *in vivo* injection experiments to assess their effects on joint tissues in a rat model of OA. To examine the functional role of STING in OA pathophysiology-particularly its involvement in IPFP-MDV-mediated inflammatory cascades. To test this, we first established an experimental model of OA in 8-week-old male Sprague-Dawley rats via intra-articular injection of MIA to induce OA. Four weeks after induction, PBS or MDVs with or without the selective STING antagonist, SN011, were administered via intra-articular injection. The rats were euthanized after two weeks, and articular cartilage tissues were harvested for further analysis (Fig. 8A).

Radiographic and micro-CT analyses revealed that OA-MDV injection significantly exacerbated joint destruction and osteophyte formation in the OA rat model, whereas SN-011 treatment markedly attenuated these structural abnormalities (Fig. 8B–D). Quantitative analyses showed that joint architecture progressively deteriorated in the OA-MDV group, and STING pathway inhibition provided protection (Fig. 8C–E). According to the results of H&E staining, the Mankin score was used to determine the degree of synovial inflammation in the knee joints of rats in each group. Scoring according to the cartilage structure density, number of chondrocytes, and the cartilage matrix staining. Synovial inflammation of the OA-MDV group was more serious and there were more serious changes such as the destruction of cartilage structure, fewer chondrocytes, less matrix staining. WT-MDV group displayed synovitis scores comparable to those of the OA group. SN011 treatment successfully reversed the elevated inflammation caused by OA-MDV (Fig. 8B and C). Safranin O/Fast Green staining was performed to examine cartilage degradation and ECM loss. Both OA-MDV and WT-MDV groups showed obvious destruction of articular cartilage, with more severe damage in the OA-MDV group. SN-011 substantially relieved OA-MDV -induced inflammatory harm. Histological analysis showed that the highest OARSI score was found in OA-MDV group indicating that MDV was a factor causing OA-related cartilage pathology in rats (Fig. 8D and E). IHC analysis showed a significant decrease of aggregated proteoglycan expression in both MDV groups, and the greatest decrease and difference from all other groups was in the OA-MDV group. OA-MDV + SN-011 group proteoglycan level was restored. However, Sting protein expression was the opposite, which had the significantly increased protein expression in both MDV groups and the highest level in OA-MDV. Elevation was reduced after treatment with SN-011 (OA-MDV + SN-011), which supports *in vivo* evidence that MDVs promote cartilage matrix damage during OA progression through the cGAS-STING signaling pathway. (Fig. 8F–I). Similarly, Sox9 expression was reduced in the cartilage tissues from all MDV-treated groups, with the greatest reduction found in the OA-MDV group (Fig. 8J and K). To verify these results on a transcriptional level, qPCR was done. Aggrecan and Sox9 mRNA expression levels were reduced in all MDV groups, and the OA-MDV group had the most significant reduction. and the downregulation was attenuated by SN-011 as expected by the protein expression (Fig. 8L–N). Together, these results indicate that WT-MDVs and OA-MDVs promote articular cartilage degradation and worsen OA severity, and the OA-MDV group has a stronger effect. These results combined with protein microarray analyses suggest that the pro-inflammatory activity of MDVs mainly occurs via cGAS-STING signaling.

### 3.9 OA-MDVs Exacerbate Osteoarthritis via cGAS-STING-associated inflammation, matrix degradation, and chondrocyte apoptosis

**Fig. 9:**
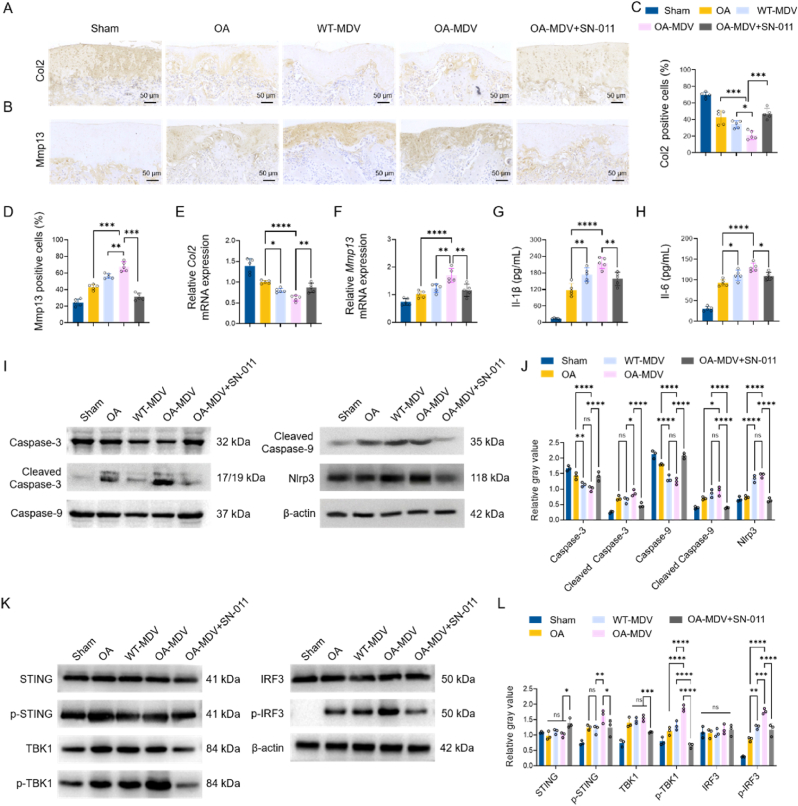
OA-MDVs Exacerbate Osteoarthritis via cGAS-STING-Associated Inflammation. (A) IHC staining of Col2 in articular cartilage. (B) IHC staining of Mmp13 in articular cartilage. (C) Quantification of Col2-positive cells (n = 5). (D) Quantification of Mmp13-positive cells (n = 5). (E–F) qPCR analysis of *Col2*, *Mmp1*3 mRNA levels (n = 5). (G–H) Quantification of Il-1β and Il-6 using ELISA (n = 5). (I) WB analysis of Caspase-3, Cleaved Caspase-3, Caspase-9, Cleaved Caspase-9, Nlrp3, and β-actin. (J) Relative gray value of Caspase-3, Cleaved Caspase-3, Caspase-9, Cleaved Caspase-9, Nlrp3 (n = 3). (K) WB analysis of STING, p-STING, TBK1, p-TBK1, IRF3, p-IRF3 and β-actin. (L) Relative gray value of STING, p-STING, TBK1, p-TBK1, IRF3, p-IRF3 (n = 3). ∗*p* < 0.05, ∗∗*p* < 0.01, ∗∗∗*p* < 0.001, and ∗∗∗∗*p* < 0.0001.

To further confirm that the cGAS-STING pathway is involved in the pathogenic effects of MDVs on cartilage ECM metabolism, oxidative stress and apoptosis-related signal pathways in the murine OA model, the following experiments were performed. Immunohistochemical staining (Fig. 9A and B) and quantitative analysis (Fig. 9C and D) indicated that there were less Col2 positive cells and more Mmp13 expression in WT-MDV and OA-MDV group compared to the normal group, with the most severe degradation observed in OA-MDVs. Quantitative PCR analysis (Fig. 9E and F) also showed a similar result on the transcription level. Col2 mRNA decreased and Mmp13 mRNA increased in the OA and WT-MDV group with the worst result in the OA-MDV group. These changes are significantly better with cGAS STING inhibitor, SN-011 both at the protein as well as RNA level. Furthermore, a strong inflammatory reaction occurred, with a considerable increase in the production of Il-6 and Il-1β after OA-MDV (Fig. 9G and H) was given, and this was noticeably reduced by SN-011. WB analysis and quantitative analysis (Fig. 9I and J) showed that there was an increase in the expression of apoptotic pathway related protein, the cleaved caspase-3, cleaved caspase-9 and Nlrp3 were up-regulated in the OA-MDV group. SN-011 treatment largely put the brakes on those pro-apoptotic and inflammatory mediators. Injection of OA-MDV was able to activate the STING signaling pathway as detected by WB, which showed the increased phosphorylation of STING, TBK1 and IRF3 *in vivo*. This activation was significantly inhibited by SN-011 treatment, as confirmed by quantitative analysis showing a significant inhibition of the OA-MDV-induced STING pathway activation (Fig. 9K). Quantitative analysis confirmed that SN-011 greatly suppressed the OA-MDV induced pathway activation (Fig. 9L). These data collectively indicate that OA-MDVs aggravate ECM degradation, oxidative stress and apoptosis of chondrocytes mainly via the cGAS-STING pathway, which can be effectively alleviated by SN-011.

## 4 Discussion

EVs significantly contribute to osteoarthritis pathogenesis by intercellular communication and pathological matrix remodeling via activation of inflammatory pathways [34]. These membrane bound vesicles are able to change joint tissues by getting rid of the ECM [35], modulating inflammation [36], facilitating angiogenesis [37], and contributing to antigen presentation [38]. For instance, EVs derived from IL-1β-activated fibroblast-like synoviocytes promote chondrocyte degeneration manifesting osteoarthritis-like pathological phenotypes [39]. EVs propagate inflammation and cartilage degradation through systemic delivery and enzymatic potentiation of inflammatory mediators and matrix-degrading proteases [40]. They also induce synovial cells and chondrocytes to express OA-associated genes [41,42], including elevated expression of MMPs and a disintegrin and metalloproteinase with thrombospondin motifs [43], which are key enzymes in cartilage and ECM degradation.

Additionally, cell-derived EVs released into the inflammatory synovial fluid of patients with OA are believed to play a pivotal role in disease progression [44,45]. These EVs have been implicated in initiating and propagating inflammatory responses as well as contributing to tissue degeneration [46]. In this study, we identified MDVs as a subpopulation of EVs that are significantly elevated in synovial fluid-though not the most abundant-supporting the hypothesis that MDVs may be closely associated with the OA progression. Common EV subpopulations such as apoptotic bodies and platelet-derived vesicles-biomarkers including C1QC and CD42a-were not detected in the synovial fluid, and therefore were not further investigated. While the intracellular roles of MDVs, such as maintaining mitochondrial quality control [47,48], mediating damage-associated molecular patterns [49], and participating in mitochondrial antigen presentation [50], are well established, their extracellular functions remain largely unexplored. A few studies suggest that MDVs may act as metabolic regulators with partial mitochondrial function[[51], [52], [53]]. However, more evidence points to MDVs functioning as immune activators, such as those inducing excitotoxicity in cardiomyocytes or activating the BCL2 pathway in target cells. Given the known pathways by which mitochondria trigger intracellular inflammatory signaling, we propose that MDVs may carry bioactive molecules such as SMAC, N-formyl peptides, cardiolipin, Cytochrome C, and mtDNA [54], all of which have the potential to activate inflammatory responses. Among these targets, we focused on the mtDNA-mediated cGAS-STING pathway, as it is directly involved in the production of inflammatory cytokines and is closely linked to the pathological progression of OA [55]. Our experiment shows that the EVs from OA patients have more free mtDNA compared to the normal ones, and the more mtDNA, the more TOMM20 carried by the EVs. It supports our hypothesis that MDVs contribute to inflammation through the mtDNA-cGAS-STING signaling axis, which supports our proposal. Isolation Protocol yielded MDVs with an estimated purity >70 % in the 20 % iodixanol fraction. Though this is enough for the functional analysis of these proteins, we can increase the purity of the subpopulation of interest if we couple density gradient centrifugation with an immunoaffinity approach. The cultured cells were then checked with IF and flow cytometry to see that they had a lot of stem cell markers like CD105 and CD73 and no blood-making or white blood cell markers like CD34 and CD45 (Fig. 3B and C). NanoFCM profiling of synovial EVs identified disease-specific vesicle subpopulations in OA. Expansion of CD63+TOMM20+ double-positive population in OA samples points at MDVs being active inflammatory vectors, which fits with our biochemical data indicating mtDNA enrichment in OA-EVs. MDV expansion points towards mitochondria stress responses being conveyed via lysosomal crosstalk (as suggested by CD63 coexpression) to transport mitochondrial cargo (TOMM20+) and expose mtDNA to cytoplasmic sensors like cGAS. Such vesicular trafficking positions MDVs as specialized mediators that link mitochondrial dysfunction to synovial inflammation, rather than passive debris. Furthermore, the progressively increased MDVs across different OA severities also imply that MDVs play a role in enhancing inflammatory cascade through the mtDNA-STING pathway. Future studies need to figure out if MDVs picky choose oxidized mtDNA fragments or just happen to have bits of broken down mitochondria, so that they could better tell if MDVs are good for telling how bad OA is or helping to make OA better.

This study utilized the GSE216651 Single Cell Sequencing Dataset in comparing the cellular heterogeneity, MDV related gene expression in joint tissue among healthy and OA patients. The Split-UMAP analysis showed that the OA pathology maybe associated with specific cell subpopulations. Among various cell subtypes, IPFP-MSCs exhibited higher scores for MDV formation-related genes compared to other subsets, providing a potential basis for their role as a source of MDVs. However, the interpretation of the cellular origin of MDVs may also be influenced by the tissue types covered and the gene scoring methods used in the single-cell sequencing data. Notably, *RAB9A* and *SNX9* expression in IPFP-MSCs was significantly higher in the WT group than in the OA group, whereas *VPS35* expression was elevated in the OA group. These findings are consistent with those of previous studies showing that *VPS35* regulates mitochondrial fission-fusion balance by modulating DLP1 complex turnover and that its dysfunction contributes to mitochondrial quality defects-an established mechanism in neurodegenerative disorders such as Parkinson's disease [56]. The predominant expression of *RAB9A* and *SNX9* in WT cells may reflect their roles in maintaining cellular homeostasis, whereas their downregulation in OA could impair the efficiency of MDV biogenesis. Notably, the elevated *VPS35* expression in OA IPFP-MSCs may indicate a compensatory mechanism involving MDV hypersecretion in response to mitochondrial dysfunction. This is in line with previous reports showing VPS35-driven overproduction of peroxisome-targeted MDV in neurodegeneration [57]. qPCR validation supported the reliability of the sequencing data. Collectively, these results suggest that OA-derived IPFP-MSCs may extensively secrete MDVs via VPS35-mediated mechanisms; however, the functional consequences of this process require further exploration.

Although mitochondrial dysfunction in chondrocytes and the anaerobic metabolism tendency of M1 macrophages in OA have been reported [58,59], we chose to focus on IPFP-MSCs for several reasons. We decided for these two reasons. First off, this direction of work builds upon the previous work [26]. Second, the effects of EXOs produced by these cells on OA have become more recognized in recent years [60,61]. Identification of a different type of EVs-MDVs-from IPFP-MSCs adds to the field. IPFP is a singular adipose tissue reservoir within the joint capsule and near to the synovium with whom it has direct contact [62]. Its main purpose is to distribute the synovial fluid and spread out the mechanical force in the knee. The IPFP being anatomically close to periarticular structures is involved in OA progression. Previous studies show that IPFP is a double-edged sword in OA, which promotes inflammation and aggravates the degree of OA [63]. This phenomenon might be due to different regulatory effects of various EV populations released by IPFP-MSCs. The concentrations and mtDNA content of WT-MDV are lower than those of OA-MDV. From proteomic analysis, it is found that WT-MDVs contain many mitochondrial matrix components, such as respiratory chain proteins, and OA-MDVs contain more mitochondrial outer membrane components, cytochrome C and mtDNA-related substances. But we don't know how these different MDV types end up with such different makeups. It is generally thought that mtDNA release can lead to an increase of the inflammatory cytokines. So the way that OA-MDV contribute to the progression of osteoarthritis probably has a lot to do with how the proteins inside them are different from each other. Despite the impairment on the formation of cartilage pellets and the contribution to the damages of articular cartilage, both types of MDVs (in the same concentration) could contribute to the formation of cartilage pellets and promote the damages of articular cartilage. The low level of the abundance of WT-MDVs may facilitate their clearance and preserve the homeostasis of the joint cavity's EV microenvironment.

Previous studies have indicated that cGAS-STING is activated in OA mainly because of the released nuclear DNA caused by mechanical stress [64,65]. However, it is not known whether exogenous mtDNA can directly induce cGAS activation. Additionally, mitochondrial calcium overload, oxidative stress as well are in the position to cause the release of mtDNA [66,67]. Therefore, the activation of cGAS-STING through mtDNA delivery by EVs is a new perspective. , which leads to the phosphorylation of IRF3 and IκB kinase (IKKs), resulting in the expression of type [68,69]. In this study, we employed a protein microarray to assess 181 key proteins associated with inflammatory responses and observed upregulation consistent with activation of the cGAS-STING pathway, with KEGG enrichment in the cytosolic DNA-sensing pathway. In addition, a subset of inflammatory markers was quantified using ELISA for validation. We hypothesize that activation of the cGAS-STING signaling pathway is driven by mtDNA release from IPFP-MDVs in OA. This mtDNA release may trigger cGAS, promoting cGAMP production and subsequent STING pathway activation. By inhibiting vesicular endocytic pathways, we found that OA-MDV tends to enter target cells through direct fusion, increasing the risk of mtDNA delivery into the cytoplasm. Therefore, we speculate that OA-MDV activation of cGAS-STING may be significantly associated with its internalization pathway. WT-MDV is more likely to enter via endocytosis, which is often lead to lysosomal degradation, and may prevent mtDNA from being recognized by cGAS in the cytoplasm. However, the deeper causes and impacts of these different internalization pathways for the two MDVs were not further explored in this study.

It has been reported that inflammatory cytokines involved in OA progression, such as IL-1β, can also activate cGAS-STING signaling [70]. further supporting the association between the cGAS-STING cascade and OA pathogenesis. In OA, fibroblast-like synoviocytes (FLS) exhibit tumor-like behaviors, contributing to persistent joint inflammation and tissue destruction [71]. Moreover, IL-1β has been shown to induce DNA damage and upregulate STING expression in a time- and dose-dependent manner [72]. This activation promotes the migration and invasion of FLS, key contributors to the aggressive phenotype of synovial tissue in OA. STING activation causes a strong pro-inflammatory reaction through NF-κB signaling, which leads to the production of cytokines like TNF-α and IL-6 [73]. These results were confirmed by the results of our protein microarrays, qPCR, and ELISAs. Interestingly, both WT-MDVs and OA-MDVs exacerbated the chondrocyte damage via activation of cGAS-STING inflammatory pathway. To better understand the downstream mechanism, we did some extra experiments showing that MDVs can also lead to mitochondrial-dependent cell death and the activation of inflammasomes. MDV treatment caused cytochrome C release, Caspase-9 and Caspase-3 activation and cleavage of these executioner caspases, indicating engagement of the intrinsic pathway. Also, concurrent upregulation of Nlrp3 indicates that there is crosstalk between inflammatory and apoptotic signaling pathways, thus indicating the multi-functional nature of MDVs in causing joint destruction. These results place MDVs at the center of OA pathology, acting as potent amplifiers of inflammation and tissue damage via cGAS-STING and related pathways. Interestingly, the differentially activated pathways such as NF-κB by WT-MDVs and OA-MDVs could be due to different cargos. And though OA-MDVs prefer to use the cGAS-STING route via mtDNA, WT-MDVs may go other ways, so we need to look at each type in its own way when we do our research later. It is important to note that the dual function of MDVs-as both drivers of pathology in OA and as natural STING agonists-has the potential to affect a wide range of other diseases, not only OA, but also antitumor immunotherapy. The present study indicates that MDVs harboring mtDNA-most likely produced by IPFP-MSCs under VPS35 control - are pathological participants in OA progression. Thus therapeutic and preventative efforts can be pursued on at least three complementary fronts:(i) attenuate the generation of MDVs such as by targeting VPS35,(ii) block MDV membrane fusion with chondrocytes, and (iii) attenuate downstream STING pathway activation. Our injection concentration (12 μg/mL) was directly extrapolated from human OA synovial fluid measurements, ensuring it reflects endogenous pathological levels rather than artificial overexpression.

There are some limits to this study. We first looked straight at IPFP-MSCs as a source of MDVs. Although scRNA-seq data offer support, it's tricky to pinpoint exactly which cell types release MDVs into synovial fluid. Secondly, the lack of commercially available VPS35 inhibitors limited our ability to validate the origin of MDVs. Additionally, while the concentration of WT-MDVs and OA-MDVs in synovial fluid varied, they displayed similar functional activity in promoting inflammation. In particular, because of the lack of standardization of mtDNA level within the MDVs, there would be possible ommissions. Further integration of mtDNA genomics and metabolomics profiling is warranted to clarify the composition and potential functions of MDVs.

## 5 Conclusion

We found that MDVs, a subpopulation of EVs, are significantly elevated in osteoarthritis synovial fluid where IPFP-MSCs constitute their primary cellular source. Both *in vitro* and *in vivo* experiments demonstrate that MDVs activate the cGAS-STING signaling pathway in recipient cells, triggering inflammatory cascades that accelerate OA pathogenesis. This study not only elucidates the pathological function of MDVs in osteoarthritis progression but also expands our understanding of IPFP-MSC-derived EV mechanisms, particularly revealing a novel EV-mediated pathway for cGAS-STING activation.

## CRediT authorship contribution statement

**Shiyu Li:** Writing – review & editing, Writing – original draft, Resources, Conceptualization. **Zi Yan:** Writing – original draft, Visualization, Methodology, Data curation. **Xinwang Zhi:** Writing – original draft, Visualization, Methodology, Formal analysis, Data curation. **Weihan Zheng:** Writing – original draft, Visualization, Methodology, Data curation. **Ziqi Zhang:** Methodology, Formal analysis, Data curation. **Zhenning Dai:** Visualization, Methodology. **Wanying Chen:** Writing – review & editing, Validation. **Hui Lu:** Writing – review & editing, Validation. **Ziyi Feng:** Writing – review & editing, Validation. **Ting Cheng:** Writing – review & editing, Validation. **Wenhui Liu:** Writing – review & editing, Methodology. **Baoyu Sun:** Writing – review & editing, Methodology. **Yuhai Ma:** Writing – review & editing, Methodology. **Bing Zhang:** Supervision, Resources, Funding acquisition, Data curation, Conceptualization. **Jianyuan Zhao:** Supervision, Resources, Project administration, Funding acquisition, Data curation, Conceptualization. **Han Liu:** Writing – original draft, Validation, Supervision, Resources, Funding acquisition, Data curation, Conceptualization. **Jiacan Su:** Writing – original draft, Resources, Funding acquisition, Formal analysis, Data curation, Conceptualization.

## Ethics approval and consent to participate

The studies involving human participants were approved by the Institutional Review Board of Guangzhou Women and Children's Medical Center (Approval No. [2024]429A01) with the informed consent of the participants and comply with the Declaration of Helsinki. All animal care and experimental protocols were approved by the Institutional Animal Care and Use Committee of the Clinical Research and Animal Experimentation Ethics Committee of the Guangzhou Women and Children's Medical Center (Approval No. [2024]02006).

## Funding

This work was supported by the National Natural Science Foundation of China (82172098 to JS, 82300018 to SL, 82202344 to HL), Fundamental Research Funds for the Central Universities (21624220 to SL), GuangDong Basic and Applied Basic Research Foundation (2025A1515012604 to SL), Guangzhou Health Science and Technology General Guidance Project (20241A011044 to XZ), Jiangsu Province Natural Science Foundation project (BK20241808 to HL), Fujian Province Natural Science Foundation project (2024J01221 to HL), Shanghai Committee of Science and Technology Laboratory Animal Research Project (23141900600 to JS) and Jiangsu Province Youth Science and Technology Talent Support Project (JSTJ-2024-097 to HL).

## Declaration of competing interest

The authors declare that they have no known competing financial interests or personal relationships that could have appeared to influence the work reported in this paper.
