# Supplementary material for: Extracellular vesicles from IPFP-MSCs trigger osteoarthritis by transferring mtDNA
Source: Bioact Mater. 2025 Dec 11;58:252–73. doi: 10.1016/j.bioactmat.2025.11.046 (PMC12754226; doi:10.1016/j.bioactmat.2025.11.046)
Supplement: Multimedia component 1 [file mmc1.docx]

**Supplement information**

**Extracellular Vesicles from IPFP-MSCs Trigger Osteoarthritis by Transferring mtDNA**

Table S1. Baseline Characteristics of the Study Participants

| **Clinical Characteristics** | | **WT (n = 8)** | **OA (n = 21)** | **P-value** |
| --- | --- | --- | --- | --- |
| **Gender [n (%)]** |  | |  | 0.218 |
| Male | 5 (62.5%) | | 7 (33.3%) |  |
| Female | 3 (37.5%) | | 14 (66.7%) |  |
| **Age (years) [mean ± SD]** | 35.1 ± 4.3 | | 73.6 ± 8.8 | P < 0.001 |
| **Pain severity from OA [n (%)]** |  | |  | - |
| Mild | - | | 8 (38.1%) |  |
| Mod/Severe | - | | 13 (61.9%) |  |
| **OA Grade [n (%)]** |  | |  | - |
| 1 | - | | 0 (0%) |  |
| 2 | - | | 6 (28.6%) |  |
| 3 | - | | 8 (38.1%) |  |
| 4 | - | | 7 (33.3%) |  |

*Pain severity: A 10-levels Numerical rating scales, categorized into ordinal groups (1-3: Mild; 4-6: Moderate; 7-10: Severe).*

*P-values calculated using Chi-square test for categorical variables and Fisher's exact test for continuous variables.*

Table S2. Antibodies used in Article

| Antibody | Dilution | Catalog Number | | | Supplier |
| --- | --- | --- | --- | --- | --- |
| **Primary antibodies (Rabbit lgG)** | | | | | |
| CD63 | WB: 1/1000 | AF1471 | | Beyotime | |
| CD81 | WB: 1/1000-1/2000 | AF2428 | | Beyotime | |
| CD9  CD105  CD73  CD45 | WB: 1/1000  IF: 1/1000; FC: 1/1000  FC: 1/50  FC: 1/200 | AF0108  ab2529  ab317462  D9M8I | | Beyotime  Abcam  Abcam  CST | |
| PDH | WB: 1/1000 | 2784S | | CST | |
| HSP60 | WB: 1/1000 | | ab190828 | Abcam | |
| TOMM20 | WB: 1/1000; FC: 1/90 | ab186735 | | Abcam | |
| VDAC1  Aggrecan  Collagen II  SOX9 | WB: 1/500  WB: 1/200  IF: 1/500; IHC: 1/200  IF: 1/1000; IHC: 1/500 | HY-P80369  ab36861  ab34712  ab185966 | | MCE  Abcam  Abcam  Abcam | |
| Caspase-3  Cleaved Caspase-3  Caspase-9  Cleaved Caspase-9  NLRP3  MMP13 | WB: 1/500  WB: 1/500  WB: 1/1000  WB: 1/1000  WB: 1/1000  IHC: 1/100 | 19677-1-AP  25128-1-AP  ab32539  Asp315  ab263899  ab315267 | | Proteintech  Proteintech  Abcam  CST  Abcam  Abcam | |
| Aggrecan  STING | IHC: 1/2000  WB: 1/1000; IHC: 1/4000 | ab313636  ab239074 | | Abcam  Abcam | |
| p-STING | WB: 1/1000 | 39887S | | CST | |
| TBK1 | WB: 1/4000 | 83686-3-RR | | Proteintech | |
| p-TBK1 | WB: 1/1000 | 82382-2-RR | | Proteintech | |
| IRF3 | WB: 1/10000 | 11312-1-AP | | Proteintech | |
| p-IRF3 | WB: 1/2000 | 29528-1-AP | | Proteintech | |
| **Primary antibodies (Mouse lgG)** | | | | | |
| Anti-C1QC | WB: 1/1000 | ab75756 | | Abcam | |
| Anti-CD42a  Anti-CD34 | WB: 1/1000  FC: 1/50 | ab173503  ab8536 | | Abcam  Abcam | |
| Anti-β-actin | WB: 1/1000 | PA1-183 | | Invitrogen | |
| **Secondary antibody (Goat IgG)** | | | | | |
| Goat anti-Rabbit IgG (H+L) | 1/5000 | 31466 | | Invitrogen | |
| Goat Anti-Mouse IgG H&L | 1/10000 | ab205719 | | Abcam | |

*IF: Immunofluorescence; IHC: Immunohistochemistry; WB: Western Blot; FC: Flow Cytometry.*

Table S3. Gene primer sequences for qPCR

| Gene | Direction | Primer sequences |
| --- | --- | --- |
| *ACAN* | Forward | GTGGAGCCGTGTTTCCAAG |
|  | Reverse | AGATGCTGTTGACTCGAACCT |
| *Collagen II* | Forward | TGGACGATCAGGCGAAACC |
|  | Reverse | GCTGCGGATGCTCTCAATCT |
| *SOX9* | Forward | AGTACCCGCATCTGCACAAC |
|  | Reverse | ACGAAGGGTCTCTTCTCGCT |
| *SOX5* | Forward | CAGCCAGAGTTAGCACAATAGG |
|  | Reverse | CTGTTGTTCCCGTCGGAGTT |
| *SOX6* | Forward | GGATGCAATGACCCAGGATTT |
|  | Reverse | TGAATGGTACTGACAAGTGTTGG |
| *RUNX2* | Forward | TGGTTACTGTCATGGCGGGTA |
|  | Reverse | TCTCAGATCGTTGAACCTTGCTA |
| *COL10A1* | Forward | ATGCTGCCACAAATACCCTTT |
|  | Reverse | GGTAGTGGGCCTTTTATGCCT |
| *PRG4* | Forward | AAAGTCAGCACATCTCCCAAG |
|  | Reverse | GTGTCTCTTTAGCGGAAGTAGTC |
| *COMP* | Forward | GATCACGTTCCTGAAAAACACG |
|  | Reverse | GCTCTCCGTCTGGATGCAG |
| *GAPDH* | Forward | GTCTCCTCTGACTTCAACAGCG |
|  | Reverse | ACCACCCTGTTGCTGTAGCCAA |
| *STING* | Forward | CCAGAGCACACTCTCCGGTA |
|  | Reverse | CGCATTTGGGAGGGAGTAGTA |
| *IL-1**β*  *β-globin*  *ND1* | Forward | GAAATGCCACCTTTTGACAGTG |
|  | Reverse  Forward  Reverse  Forward  Reverse | TGGATGCTCTCATCAGGACAG  AAAGGTGCCCTTGAGGTTGTC  TGAAGGCTCATGGCAAGAAA  CCCTAAACCCGCCACATCT  GAGCGATGGTGAGAGCTAAGGT |
| *ND2* | Forward  Reverse | CCCACACACACACCGCTAAC  ATTGCTTTCGTGGTTATCTGCT |
| *COX1* | Forward  Reverse | TCGCCGACCGTTGACTATTCT  ATGAGCCGCAGGTACTGTATC |
| *ATP6*  *MMP13*  *Aggrecan*  *Col2a1*  *Sting1*  *Sox9*  *Mmp13*  *Gapdh* | Forward  Reverse  Forward  Reverse  Forward  Reverse  Forward  Reverse  Forward  Reverse  Forward  Reverse  Forward  Reverse  Forward  Reverse | ACCAACACCTCTTTACAGTGAA  TGGTTGTCCTGCGCTATCTG  TCCCAGGAATTGGTGATAAAGTAGA  CTGGCATGACGCGAACAATA  TCATTCTCCGCGGCTAGCAT  TCGATGGAGGGAAGGGACAC  CATCGAGTACCGATCACAGAAG  GCCCTATGTCCACACCAAAT  TCAGTGGTGCAGGGAGCCGA  CGCCTGCTGGCTGTCCGTTC  AGCCCTGGTTTCGTTCTCTG  AGGAGATTCATACGCGAGCC  AGCTCCAAAGGCTACAACTTAT  GTCTTCATCTCCTGGACCATAG  AAGCCCATCACCATCTTCCA  ATGGCATGGACTGTGGTCAT |

Table S4. Abbreviation

| **Abbreviation** | **Full name** |
| --- | --- |
| ADAMTS  ATP6  BCA  CAT | a disintegrin and metalloproteinase with thrombospondin motifs  ATP synthase F0 subunit 6  Bicinchoninic Acid Assay  Catalase |
| cDNA  COL2  COX1  CRP | complementary DNA  Collagen type II  Cytochrome c oxidase subunit 1  C-reactive protein |
| cGAS | Cyclic guanosine phosphate adenosine synthase |
| DAMPs | damage-associated molecular patterns |
| DEPs  DMEM  DIO  ECAR  ECM | Differential expression proteins  Dulbecco’s modified Eagle’s medium  Dioctadecyloxacarbocyanine  Extracellular Acidification Rate  extracellular matrix |
| EVs  EXOs  FLS  GO  GSEA  H&E  HRP  IF  IMM  IPFP  IPFP-MSCs  IRF3  IL-1β  IHC  IRF3  IKKs  mtDNA  MDA  MDVs  MMP13  MIA  MMPs  ND1  ND2  NF-κB  NTA  OA  OARSI  OCR  OD  OMM  OPA1  PRG4  PRKN  PS  PFA  qRT-PCR  RHOT1  SNX9  SOD  STX17  SOX9  TBK1  TEM  TNF  TOLLIP  TOMM20  VDAC1  WT | extracellular vesicles  exosomes  fibroblast-like synoviocytes  Gene Ontology  Gene Set Enrichment Analysis  Hematoxylin and Eosin  Goat anti-rabbit IgG H&L  Immunofluorescence  inner membrane of mitochondria  infrapatellar fat pad  infrapatellar fat pad mesenchymal stem cells  interferon regulatory factor 3  interleukin-1β  Immunohistochemistry  interferon regulatory factor 3  IkappaB kinase  mitochondrial DNA  Malondialdehyde  mitochondrial-derived vesicles  matrix metalloproteinase-13  iodoacetic acid  matrix metalloproteinases  NADH dehydrogenase subunit 1  NADH dehydrogenase subunit 2  nuclear factor kappa B  Nanoparticle tracking analysis  Osteoarthritis  Osteoarthritis Research Society International  Oxygen Consumption Rate  optical density  outer membrane of mitochondria  OPA1 mitochondrial dynamin-like GTPase  Proteoglycan 4  Parkin RBR E3 ubiquitin protein ligase  penicillin-streptomycin  Paraformaldehyde  quantitative reverse transcription polymerase chain reaction  Ras homolog family member T1  sorting nexin 9  Superoxide Dismutase  syntaxin 17  SRY-Box Transcription Factor 9  TANK binding kinase 1  Transmission Electron Microscopy  tumor necrosis factor  Toll-interacting protein  translocase of the outer mitochondrial membrane complex subunit 20  voltage-dependent anion channel 1  wild-type |

Table S4. List of DEPs from protein microarry analysis

| **Gene** | **WT1** | **WT2** | **Positive1** | **Positive2** | **WT-MDV1** | **WT-MDV2** | **OA-MDV1** | **OA-MDV2** |
| --- | --- | --- | --- | --- | --- | --- | --- | --- |
| POS1 | 38,888 | 48,141 | 30,713 | 45,133 | 33,631 | 44,467 | 33,247 | 31,707 |
| POS2 | 9,476 | 10,708 | 10,184 | 9,246 | 11,265 | 8,205 | 10,228 | 6,259 |
| POS3 | 7,248 | 7,793 | 8,158 | 5,289 | 8,756 | 7,102 | 7,342 | 6,655 |
| Neg | 590 | 591 | 388 | 307 | 282 | 411 | 400 | 389 |
| AIM2 | 773 | 805 | 560 | 589 | 593 | 696 | 572 | 694 |
| AKT1 | 904 | 722 | 569 | 592 | 596 | 593 | 412 | 512 |
| 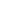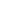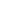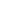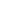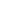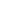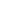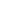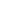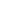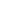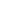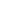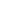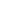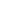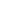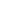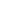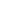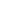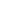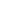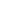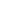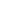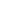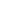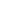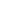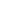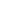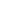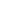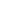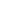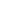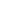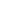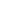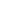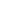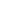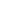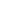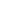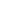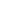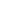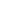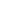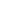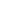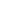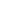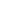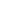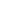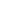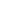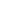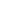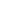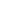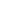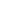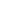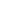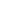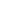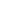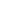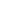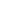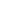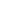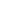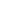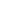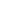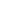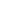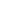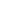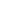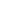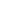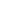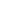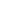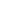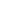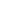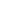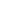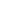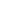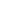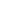AKT2 | 700 | 713 | 396 | 400 | 511 | 514 | 399 | 425 |
| AKT3 | 860 | 738 | 389 | 408 | 520 | 502 | 411 | 395 |
| ANTXR1 | 972 | 1,091 | 482 | 491 | 697 | 703 | 404 | 604 |
| ANTXR2 | 965 | 881 | 415 | 381 | 509 | 514 | 397 | 410 |
| ATG12 | 902 | 1,097 | 510 | 502 | 586 | 601 | 506 | 475 |
| ATG5 | 880 | 879 | 488 | 513 | 526 | 601 | 419 | 419 |
| BCL2 | 605 | 608 | 506 | 502 | 509 | 496 | 424 | 420 |
| BCL2L1 | 706 | 633 | 599 | 582 | 565 | 495 | 510 | 414 |
| BIRC2 | 713 | 688 | 391 | 501 | 601 | 493 | 397 | 406 |
| BIRC3 | 791 | 674 | 497 | 388 | 484 | 500 | 505 | 394 |
| CAMP | 1,902 | 1,735 | 1,971 | 1,622 | 1,825 | 1,832 | 1,920 | 1,523 |
| CARD18 | 1,006 | 916 | 839 | 809 | 795 | 866 | 820 | 697 |
| CARD9 | 709 | 698 | 507 | 507 | 596 | 606 | 499 | 621 |
| CASP1 | 593 | 607 | 394 | 394 | 512 | 500 | 411 | 505 |
| CASP4 | 11,294 | 886 | 509 | 614 | 686 | 654 | 621 | 702 |
| CASP5 | 897 | 1,002 | 583 | 592 | 688 | 689 | 600 | 706 |
| CASP8 | 1,010 | 1,104 | 738 | 781 | 993 | 1,202 | 1,000 | 1,050 |
| CASR | 1,413 | 1,215 | 892 | 811 | 881 | 790 | 767 | 867 |
| CCL2 | 1,736 | 1,626 | 847 | 822 | 1,161 | 993 | 1,020 | 1,028 |
| CCL3 | 1,017 | 996 | 536 | 618 | 691 | 699 | 410 | 507 |
| CCL3L1 | 680 | 1,002 | 506 | 584 | 703 | 786 | 507 | 588 |
| CCL4 | 586 | 612 | 498 | 474 | 613 | 590 | 507 | 387 |
| CCL4L1 | 600 | 624 | 616 | 516 | 621 | 577 | 528 | 406 |
| CCL5 | 830 | 612 | 518 | 501 | 489 | 487 | 401 | 404 |
| CD14 | 809 | 710 | 399 | 395 | 391 | 507 | 399 | 392 |
| CD40 | 581 | 670 | 501 | 490 | 706 | 506 | 387 | 510 |
| CD80 | 598 | 584 | 530 | 518 | 499 | 533 | 500 | 701 |
| CD86 | 587 | 724 | 393 | 515 | 502 | 496 | 534 | 608 |
| CHUK | 719 | 609 | 569 | 525 | 540 | 504 | 481 | 380 |
| CTSB | 1,864 | 1,143 | 616 | 585 | 709 | 711 | 872 | 894 |
| CTSK | 798 | 890 | 694 | 610 | 698 | 672 | 680 | 590 |
| CXCL1 | 779 | 788 | 526 | 500 | 602 | 600 | 511 | 399 |
| CXCL10 | 895 | 767 | 820 | 795 | 1,035 | 598 | 511 | 575 |
| CXCL11 | 863 | 1,095 | 498 | 517 | 519 | 672 | 401 | 495 |
| CXCL2 | 1,213 | 1,247 | 2,702 | 2,792 | 991 | 926 | 677 | 766 |
| CXCL3 | 605 | 592 | 500 | 501 | 522 | 589 | 397 | 504 |
| CXCL8 | 904 | 995 | 1,110 | 785 | 809 | 794 | 680 | 691 |
| CXCL9 | 891 | 873 | 921 | 924 | 758 | 595 | 581 | 612 |
| CYBA | 706 | 616 | 483 | 381 | 494 | 610 | 411 | 390 |
| DEFA1 | 576 | 681 | 498 | 487 | 512 | 514 | 404 | 399 |
| DEFA3 | 816 | 704 | 601 | 601 | 598 | 706 | 587 | 506 |
| DEFA4 | 587 | 582 | 495 | 488 | 510 | 514 | 501 | 500 |
| DEFA5 | 707 | 699 | 492 | 513 | 599 | 598 | 517 | 636 |
| DEFA6 | 690 | 780 | 489 | 500 | 499 | 590 | 563 | 626 |
| DEFB103A | 704 | 488 | 400 | 398 | 507 | 512 | 395 | 396 |
| DEFB4A | 798 | 905 | 490 | 563 | 711 | 587 | 589 | 692 |
| ERBIN | 669 | 665 | 382 | 391 | 514 | 507 | 408 | 399 |
| FADD | 813 | 671 | 406 | 414 | 595 | 518 | 413 | 389 |
| FOS | 783 | 790 | 403 | 395 | 513 | 489 | 411 | 306 |
| GABARAP | 797 | 764 | 395 | 406 | 505 | 407 | 503 | 409 |
| GABARAPL1 | 1,527 | 819 | 522 | 569 | 602 | 674 | 600 | 665 |
| GABARAPL2 | 782 | 771 | 689 | 689 | 726 | 708 | 510 | 603 |
| GBP2 | 1,240 | 1,223 | 792 | 900 | 880 | 782 | 709 | 701 |
| GPRC6A | 805 | 787 | 768 | 663 | 736 | 696 | 509 | 493 |
| GSDMD | 563 | 708 | 511 | 518 | 513 | 786 | 523 | 473 |
| HSP90AA1 | 600 | 720 | 587 | 543 | 599 | 594 | 576 | 519 |
| HSP90AB1 | 698 | 581 | 505 | 502 | 494 | 506 | 391 | 491 |
| IFNA10 | 716 | 706 | 501 | 508 | 591 | 469 | 573 | 615 |
| IFNA14 | 888 | 907 | 784 | 739 | 698 | 781 | 829 | 873 |
| IFNA16 | 785 | 618 | 508 | 477 | 596 | 600 | 510 | 580 |
| IFNA17 | 798 | 898 | 508 | 502 | 606 | 587 | 509 | 504 |
| IFNA2 | 1,009 | 978 | 697 | 785 | 862 | 817 | 699 | 711 |
| IFNA21 | 1,158 | 987 | 809 | 763 | 904 | 931 | 732 | 778 |
| IFNA4 | 1,107 | 1,110 | 596 | 690 | 879 | 773 | 589 | 573 |
| IFNA5 | 1,012 | 1,000 | 605 | 569 | 782 | 684 | 667 | 689 |
| IFNA6 | 869 | 890 | 612 | 712 | 771 | 686 | 583 | 514 |
| IFNA7 | 597 | 600 | 503 | 401 | 502 | 514 | 411 | 405 |
| IFNA8 | 8,254 | 7,447 | 8,403 | 8,974 | 7,075 | 6,659 | 6,807 | 6,957 |
| IFNAR1 | 792 | 602 | 502 | 507 | 610 | 587 | 391 | 414 |
| IFNAR2 | 892 | 899 | 588 | 685 | 607 | 619 | 604 | 594 |
| IFNB1 | 692 | 598 | 505 | 505 | 588 | 508 | 497 | 510 |
| IKBKB | 605 | 595 | 519 | 518 | 394 | 511 | 380 | 488 |
| IKBKE | 612 | 611 | 388 | 388 | 517 | 505 | 384 | 385 |
| IKBKG | 609 | 628 | 388 | 411 | 502 | 519 | 403 | 394 |
| IL12A | 815 | 617 | 511 | 396 | 611 | 610 | 574 | 574 |
| IL12B | 700 | 696 | 390 | 496 | 598 | 591 | 415 | 502 |
| IL18 | 787 | 893 | 505 | 508 | 596 | 671 | 504 | 503 |
| IL1B | 569 | 695 | 395 | 379 | 593 | 576 | 410 | 410 |
| IL6 | 989 | 937 | 882 | 812 | 988 | 859 | 789 | 702 |
| IRAK1 | 584 | 694 | 400 | 393 | 496 | 512 | 396 | 408 |
| IRAK4 | 1,049 | 1,402 | 1,016 | 1,169 | 1,016 | 1,085 | 967 | 1,021 |
| IRF3 | 593 | 578 | 406 | 404 | 501 | 497 | 407 | 408 |
| IRF5 | 600 | 592 | 522 | 507 | 510 | 501 | 409 | 306 |
| JAK1 | 907 | 796 | 576 | 578 | 679 | 584 | 490 | 595 |
| JUN | 640 | 727 | 583 | 702 | 601 | 613 | 503 | 566 |
| LBP | 918 | 876 | 1,517 | 1,384 | 895 | 778 | 781 | 848 |
| LY96 | 705 | 709 | 513 | 496 | 403 | 503 | 394 | 498 |
| MAP2K1 | 929 | 1,089 | 746 | 783 | 670 | 613 | 588 | 596 |
| MAP2K2 | 987 | 983 | 932 | 1,147 | 964 | 889 | 691 | 790 |
| MAP2K3 | 820 | 703 | 695 | 547 | 606 | 707 | 596 | 587 |
| MAP2K4 | 895 | 806 | 604 | 591 | 663 | 608 | 584 | 665 |
| MAP2K7 | 710 | 580 | 405 | 506 | 500 | 595 | 411 | 396 |
| MAP3K7 | 597 | 591 | 397 | 406 | 493 | 489 | 390 | 415 |
| TAB1 | 607 | 602 | 479 | 500 | 583 | 604 | 497 | 511 |
| MAP3K8 | 1,069 | 847 | 687 | 567 | 853 | 695 | 674 | 689 |
| MAPK1 | 875 | 879 | 588 | 595 | 679 | 652 | 606 | 550 |
| MAPK11 | 861 | 808 | 790 | 700 | 735 | 794 | 713 | 601 |
| MAPK12 | 538 | 506 | 403 | 516 | 497 | 599 | 398 | 398 |
| MAPK13 | 826 | 777 | 702 | 699 | 679 | 597 | 587 | 689 |
| MAPK14 | 1,652 | 2,490 | 1,079 | 1,065 | 1,252 | 1,082 | 1,075 | 1,096 |
| MAPK3 | 1,341 | 1,530 | 955 | 871 | 832 | 765 | 850 | 857 |
| MAPK8 | 929 | 923 | 881 | 687 | 858 | 865 | 878 | 798 |
| MAPK9 | 711 | 593 | 394 | 513 | 516 | 504 | 404 | 509 |
| MFN1 | 609 | 592 | 389 | 442 | 510 | 497 | 383 | 386 |
| MFN2 | 662 | 782 | 506 | 516 | 514 | 615 | 575 | 517 |
| MYD88 | 575 | 600 | 404 | 400 | 876 | 507 | 409 | 514 |
| NAIP | 826 | 846 | 709 | 806 | 693 | 777 | 704 | 709 |
| NAMPT | 494 | 585 | 426 | 388 | 506 | 499 | 312 | 409 |
| NFKB1 | 488 | 446 | 394 | 400 | 501 | 511 | 408 | 402 |
| NFKBIA | 499 | 599 | 387 | 509 | 510 | 506 | 396 | 412 |
| NFKBIB | 615 | 710 | 609 | 599 | 664 | 586 | 568 | 511 |
| NLRC4 | 604 | 678 | 492 | 498 | 577 | 619 | 565 | 591 |
| NLRP1 | 611 | 499 | 396 | 402 | 473 | 609 | 391 | 405 |
| NLRP3 | 738 | 604 | 465 | 396 | 414 | 479 | 402 | 404 |
| NLRP6 | 703 | 581 | 599 | 514 | 497 | 587 | 517 | 401 |
| NOD1 | 600 | 809 | 508 | 476 | 572 | 607 | 495 | 512 |
| NOD2 | 821 | 782 | 671 | 757 | 592 | 609 | 689 | 684 |
| OAS1 | 803 | 775 | 592 | 579 | 519 | 513 | 650 | 388 |
| OAS2 | 1,009 | 1,099 | 1,196 | 1,095 | 813 | 889 | 714 | 793 |
| P2RX7 | 620 | 614 | 456 | 402 | 499 | 511 | 411 | 403 |
| PANX1 | 722 | 712 | 500 | 390 | 684 | 702 | 513 | 501 |
| PIK3CA | 1,035 | 1,090 | 780 | 873 | 878 | 808 | 661 | 689 |
| PIK3CB | 871 | 893 | 664 | 593 | 856 | 756 | 675 | 598 |
| PIK3CD | 745 | 1,089 | 584 | 572 | 693 | 585 | 583 | 596 |
| PIK3R1 | 603 | 610 | 518 | 385 | 610 | 506 | 501 | 397 |
| PIK3R2 | 588 | 604 | 585 | 606 | 623 | 505 | 503 | 510 |
| PIK3R3 | 495 | 512 | 388 | 387 | 484 | 487 | 409 | 407 |
| PKN1 | 804 | 682 | 489 | 591 | 607 | 713 | 502 | 602 |
| PKN2 | 1,182 | 979 | 647 | 667 | 564 | 475 | 587 | 521 |
| PLCB1 | 573 | 599 | 504 | 489 | 511 | 404 | 393 | 384 |
| PLCB2 | 997 | 914 | 791 | 795 | 841 | 846 | 725 | 700 |
| PLCB3 | 599 | 614 | 496 | 518 | 514 | 595 | 500 | 581 |
| PLCB4 | 577 | 505 | 390 | 514 | 511 | 513 | 394 | 522 |
| PRKCD | 687 | 794 | 498 | 490 | 702 | 609 | 581 | 589 |
| PYCARD | 776 | 803 | 615 | 503 | 687 | 695 | 599 | 592 |
| RAC1 | 592 | 600 | 407 | 393 | 800 | 584 | 490 | 500 |
| RBCK1 | 702 | 705 | 518 | 524 | 671 | 600 | 593 | 587 |
| RELA | 598 | 591 | 402 | 413 | 682 | 588 | 403 | 484 |
| RHOA | 1,213 | 1,096 | 790 | 895 | 886 | 900 | 774 | 1,017 |
| RIPK1 | 600 | 774 | 574 | 592 | 688 | 610 | 579 | 571 |
| RIPK2 | 782 | 786 | 590 | 580 | 684 | 695 | 509 | 509 |
| RIPK3 | 573 | 601 | 386 | 389 | 491 | 505 | 402 | 408 |
| RNASEL | 501 | 505 | 503 | 405 | 497 | 483 | 406 | 399 |
| RNF31 | 717 | 609 | 396 | 383 | 481 | 492 | 405 | 406 |
| SHARPIN | 812 | 802 | 491 | 695 | 508 | 618 | 511 | 591 |
| SPP1 | 709 | 702 | 505 | 483 | 570 | 499 | 571 | 483 |
| STAT1 | 1,051 | 1,091 | 1,016 | 808 | 611 | 664 | 718 | 683 |
| STAT2 | 787 | 828 | 505 | 620 | 517 | 504 | 513 | 587 |
| STING1 | 600 | 693 | 392 | 382 | 490 | 395 | 388 | 510 |
| TAB2 | 683 | 609 | 404 | 398 | 599 | 515 | 508 | 514 |
| TANK | 602 | 479 | 416 | 396 | 514 | 616 | 501 | 424 |
| TBK1 | 614 | 496 | 403 | 385 | 522 | 480 | 409 | 289 |
| TICAM2 | 507 | 491 | 393 | 412 | 507 | 492 | 483 | 411 |
| TLR1 | 615 | 603 | 399 | 398 | 503 | 506 | 404 | 414 |
| TLR2 | 509 | 500 | 390 | 394 | 512 | 497 | 399 | 407 |
| TLR3 | 596 | 577 | 397 | 399 | 495 | 503 | 287 | 391 |
| TLR4 | 621 | 690 | 401 | 505 | 570 | 583 | 409 | 491 |
| TLR5 | 688 | 702 | 479 | 404 | 611 | 687 | 398 | 416 |
| TLR6 | 1,080 | 883 | 446 | 623 | 684 | 682 | 693 | 682 |
| TLR8 | 583 | 578 | 509 | 503 | 497 | 511 | 408 | 413 |
| TLR9 | 810 | 678 | 510 | 605 | 499 | 606 | 508 | 508 |
| TNF | 811 | 793 | 681 | 705 | 612 | 527 | 679 | 610 |
| TNFAIP3 | 594 | 712 | 481 | 515 | 391 | 491 | 398 | 395 |
| TOLLIP | 1,083 | 1,292 | 961 | 807 | 627 | 603 | 498 | 589 |
| TP53BP1 | 617 | 504 | 405 | 284 | 510 | 504 | 405 | 407 |
| TRAF2 | 723 | 620 | 399 | 501 | 515 | 536 | 484 | 516 |
| TRAF3 | 591 | 490 | 292 | 388 | 486 | 503 | 417 | 397 |
| TRAF5 | 624 | 610 | 402 | 400 | 502 | 591 | 400 | 409 |
| TRAF6 | 609 | 684 | 415 | 405 | 520 | 507 | 509 | 406 |
| TRPM7 | 497 | 505 | 408 | 400 | 506 | 505 | 409 | 416 |
| TXN | 8,356 | 8,600 | 4,225 | 3,897 | 6,651 | 7,373 | 6,448 | 7,159 |
| TXN2 | 505 | 506 | 287 | 405 | 509 | 507 | 414 | 407 |
| TXNIP | 881 | 808 | 820 | 807 | 760 | 692 | 712 | 508 |
| TYK2 | 690 | 698 | 492 | 402 | 525 | 502 | 410 | 399 |
| VDAC1 | 646 | 767 | 525 | 500 | 668 | 691 | 603 | 497 |
| VDAC2 | 707 | 681 | 387 | 508 | 509 | 508 | 503 | 513 |
| MAVS | 699 | 789 | 511 | 497 | 528 | 500 | 405 | 491 |
| XIAP | 1,034 | 998 | 560 | 645 | 484 | 605 | 614 | 604 |
| YWHAE | 703 | 586 | 401 | 401 | 510 | 409 | 407 | 382 |

Figure S1. Western blot raw images.

Figure S2. Correlation analysis between EVs protein levels and mtDNA content. (A) Linear fitting results of TOMM20 levels with *ND1*, *ND2*, *COX1*, *ATP6* in WT-EVs (blue dots) and OA-EVs (pink dots). (B) Linear fitting results of TFAM levels with *ND1*, *ND2*, *COX1*, *ATP6* in WT-EVs (blue dots) and OA-EVs (pink dots).

Figure S3. (A) Marker information for cell subpopulation classification in single-cell sequencing. (B) Dimensionality reduction visualization results of MDVs formation related genes in the UMAP of OA group.

Figure S4. Score of MDV formation related gene expression levels within each cell subpopulation in the WT and OA group.

Figure S5. The effect of uptake inhibitors on the internalization of MDVs. (A) Schematic diagram: Inhibitors affect different pathways of MDVs internalization, with Dynasore inhibiting the clathrin-mediated endocytosis pathway, Cytochalasin D inhibiting the macropinocytosis pathway, Amiloride inhibiting the phagocytosis pathway, and Z-Phe-Phe-Phe-OH inhibiting the direct fusion pathway. (B) Fluorescence images of MDVs internalized by C28/I2 cells at 72 hours under different inhibitor treatment conditions. (C) Quantitative analysis of fluorescence intensity at 505 nm, using images of untreated group as the reference value (100%), **p* < 0.05, ***p* < 0.01, ****p* < 0.001 and ns: *p* > 0.05.

Figure S6. Original image of protein microarray.
